# Design, Synthesis, and Biological Evaluation of [1,2,5]Oxadiazolo[3,4-*b*]pyridin-7-ol as Mitochondrial Uncouplers for the Treatment of Obesity and Metabolic Dysfunction-Associated Steatohepatitis

**DOI:** 10.1021/acs.jmedchem.4c02366

**Published:** 2024-11-30

**Authors:** Mary A. Foutz, Emily L. Krinos, Martina Beretta, Stefan R. Hargett, Riya Shrestha, Jacob H. Murray, Ethan Duerre, Joseph M. Salamoun, Katrina McCarter, Divya P. Shah, Kyle L. Hoehn, Webster L. Santos

**Affiliations:** † Department of Chemistry and Virginia Tech Center for Drug Discovery, Virginia Tech, Blacksburg, Virginia 24061, United States; ‡ School of Biotechnology and Biomolecular Sciences, University of New South Wales, Kensington, NSW 2033, Australia; § Departments of Pharmacology and Medicine, University of Virginia, Charlottesville, Virginia 22908, United States

## Abstract

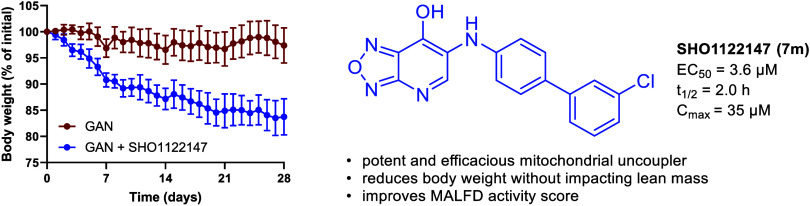

Mitochondrial uncouplers are small molecule protonophores that act to dissipate the proton motive force independent of adenosine triphosphate (ATP) synthase. Mitochondrial uncouplers such as BAM15 increase respiration and energy expenditure and have potential in treating a variety of metabolic diseases. In this study, we disclose the structure–activity relationship profile of 6-substituted [1,2,5]oxadiazolo[3,4-*b*]pyridin-7-ol derivatives of BAM15. Utilizing an oxygen consumption rate assay as a measure of increased cellular respiration, **SHO1122147** (**7m**) displayed an EC_50_ of 3.6 μM in L6 myoblasts. Pharmacokinetic studies indicated a half-life of 2 h, *C*_max_ of 35 μM, and no observed adverse effects at 1,000 mg kg^–1^ dose in mice. In a Gubra-Amylin (GAN) mouse model of MASH, **SHO1122147** was efficacious in decreasing body weight and liver triglyceride levels at 200 mg kg^–1^ day^–1^ without changes in body temperature. These findings indicate the potential of utilizing novel [1,2,5]oxadiazolo[3,4-*b*]pyridin-7-ol mitochondrial uncouplers for treatment of fatty liver disease and obesity.

## Introduction

Obesity affects 42% of people in the United States and is defined as an accumulation of excess body fat as a result of a higher influx of calories versus energy expended.^1^ Obesity is a chronic disease with multiple comorbidities including cardiovascular disease,^2^ type 2 diabetes,^3^ and nonalcoholic fatty liver disease.^4^ The disease can place excess stress on joints and drastically impact a patient’s physical activity.^5^ Not only is quality of life severally impacted, but in the United States alone, the yearly medical cost of obesity is estimated to be $173 billion.^6^

Obesity is defined by having a body mass index (BMI) score of 30 or above. Reducing caloric intake and increasing exercise are the primary means of maintaining a BMI in a healthy range. However, patient compliance with lifelong voluntary food restriction or exercise is poor, which makes healthy weight maintenance challenging.^7^ An effective treatment option is bariatric surgery; however, the procedure is not a global solution due to high cost and risk.^8^

In contrast, pharmaceutical treatments with low adverse effects represent a solution with potential high compliance. The glucagon-like peptide 1 (GLP-1) receptor agonist semaglutide is an injectable treatment for type-2 diabetes.^9^ It was approved by the FDA for weight loss in 2021, quickly becoming a popular option for obesity treatment as it leads to decreased appetite and food intake.^10^ In 2023, the FDA also approved tirzepatide for weight loss. Tirzepatide activates both GLP-1 and glucose dependent insulinotropic polypeptide (GIP).^11,12^ While both treatments are effective, undesirable side effects such as adverse gastrointestinal complications, including moderate to severe diarrhea, nausea and vomiting are observed.^13^ In STEP-1 clinical trials, semaglutide resulted in up to 40% loss of lean mass.^14^ Semaglutide and tirzepatide also have a boxed warning for increased risk of medullary thyroid carcinoma and are linked to a risk of optic nonarthritic anterior ischemic neuropathy.^15^ Subsequently, there is significant opportunity to develop new drugs that do not decrease lean mass or cause debilitating adverse effects.

While obesity is still an overwhelming problem worldwide, the number and availability of treatment options are increasing. However, when in conjunction with comorbidities such as metabolic dysfunction-associated steatohepatitis (MASH), treatment plans become exceptionally limited.^16^ MASH is an advanced form of metabolic-dysfunction associated fatty liver disease (MAFLD) where early stage liver injury progresses to steatosis and liver inflammation that can eventually lead to cirrhosis and hepatocellular carcinoma.^17−19^ To date, MASH has only one FDA-approved pharmaceutical: resmetirom–a liver-targeted thyroid hormone receptor-β agonist.^20^ Beyond this treatment, liver transplantation is the only other option. However, obesity puts patients at a higher risk for the recurrence of liver disease following transplantation.^21,22^ Thus, finding therapies to treat MASH and reverse obesity would be extremely beneficial.

While the etiology of MASH is unknown, the progression of the disease can be attributed to production of reactive oxygen species (ROS) leading to a necro-inflammatory environment.^23−25^ Similarly, obesity is a low-grade inflammatory disease that has been linked to high levels of reactive oxygen species in adipose tissue causing oxidative stress and mitochondrial dysfunction.^26^ Beyond GLP-1 and thyroid hormone receptor-β agonists, innovative strategies geared toward reducing mitochondrial dysfunction are being explored. Specifically, mitochondrial uncouplers are of interest for the treatment of both MASH and obesity because they act as “fat-burning” molecules that reduce ROS production at their source.

**Figure 1:**
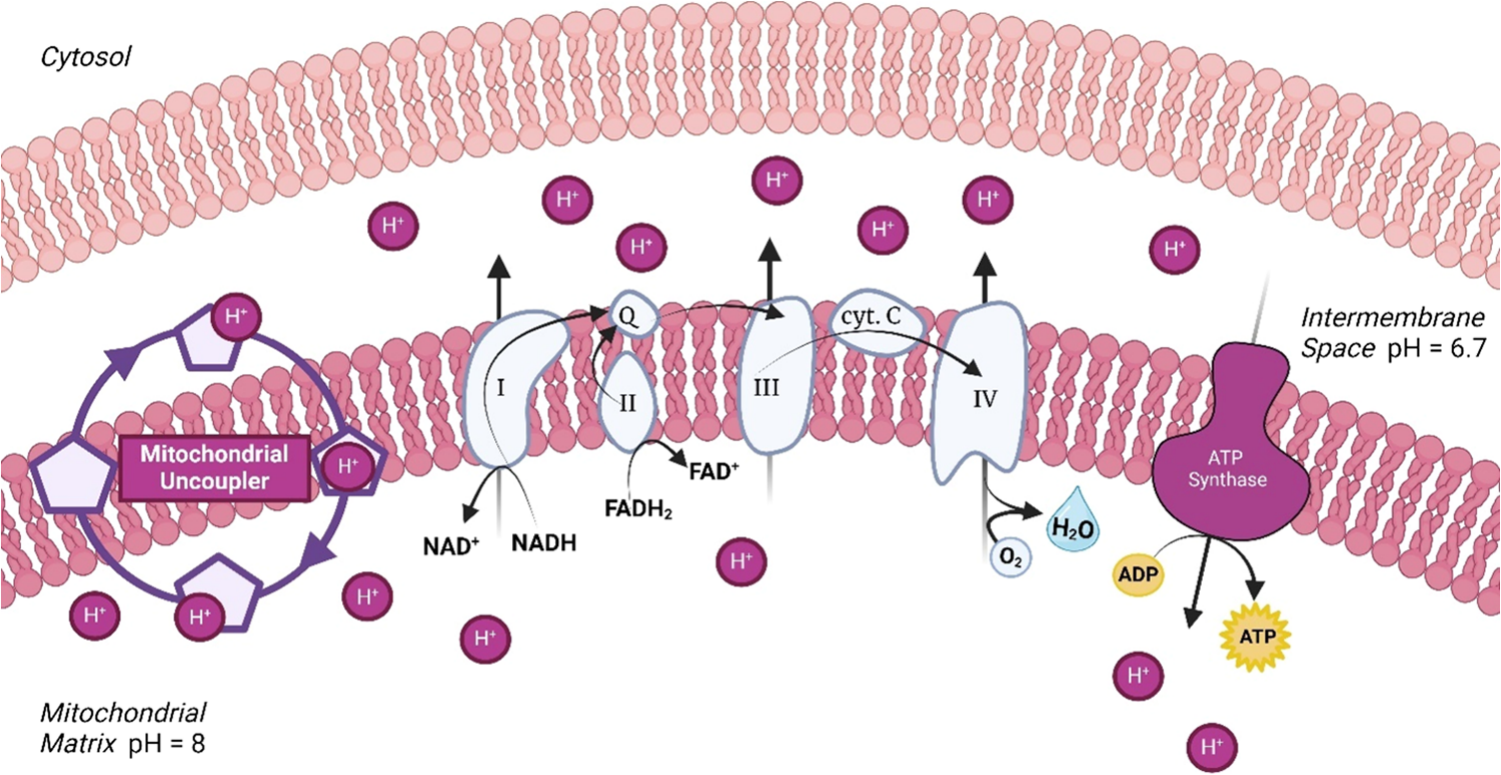
Electron transport chain established the proton motive force while mitochondrial uncouplers facilities a “proton leak”.^43^

Mitochondrial oxidative phosphorylation is used for approximately 95% of adenosine triphosphate (ATP) synthesis in most cells (Figure 1).^27^ In noninjured cells, mitochondria convert nutrients into ATP through a process that involves a proton cycle across the mitochondrial intermembrane. Nutrient oxidation in the mitochondrial matrix results in the efflux of protons into the mitochondrial intermembrane space creating a proton motive force (PMF). The resulting electrochemical gradient drives ATP synthesis as protons re-enter the matrix via ATP synthase. During this metabolic process, electrons leak from electron transport chain complexes I and III where they react with molecular oxygen to form ROS, causing inflammation.^23,24,28^ Uncoupling proteins (UCPs) induce mitochondrial uncoupling–a process where protons are transported into the matrix independent of ATP synthase, causing an increase in respiration.^29^ Efficient respiration facilitates low residence time of electrons in complex I and III, leading to a reduction of ROS formation. Thus, mitochondrial uncoupling as a potential treatment strategy for both obesity and MASH.

**Figure 2:**
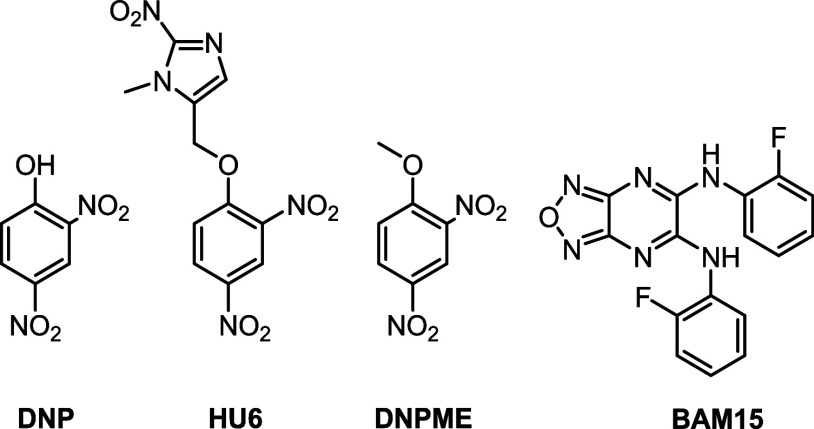
Chemical structures of select small molecule mitochondrial uncouplers.

Unfortunately, UCPs are considered undruggable targets due to their closed activation site.^30^ However, small molecules that act as proton shuttles can be utilized as alternatives to UCPs. 2,4-Dinitrophenol (DNP) was the first small molecule mitochondrial uncoupler reported where weight loss was first observed (Figure 2).^31^ However, DNP was banned by the FDA due to severe side effects such as hyperthermia, blindness, and even death.^31−34^ DNP has a narrow therapeutic window and is linked to off-target effects including depolarizing the plasma membrane and increasing intracellular calcium.^35,36^ To mitigate these issues, DNP prodrugs including liquid crystal gel (DNP LC-Gel), DNP methyl ether (DNPME), and extended release (CRMP) formulations are being explored for treatments of diabetes and MASH. Most recently, prodrug HU6 is undergoing phase II clinical trials for the treatment of MASH.^37−42^

Our lab has an ongoing interest in small molecule mitochondrial uncouplers that are structurally distinct from DNP. In 2014, we discovered BAM15 as a mitochondrial selective uncoupler.^44^ Since then, BAM15 has shown promise as a therapeutic agent for diseases such as cancer, diabetes, obesity, MASH, and sepsis.^45,46^

**Figure 3:**
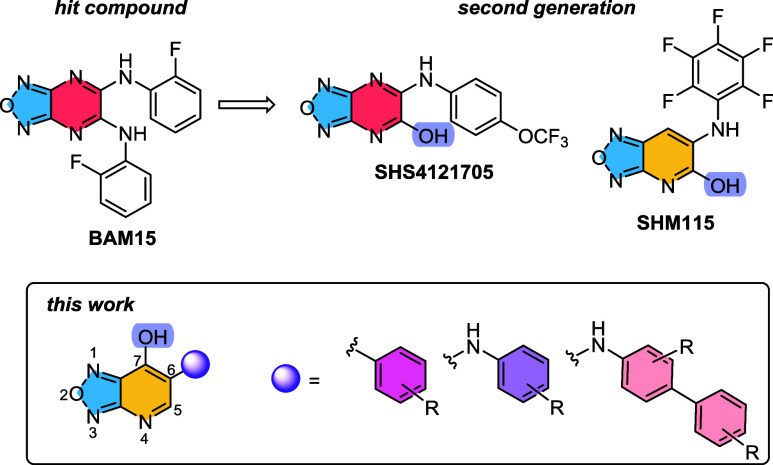
Past and current work on structure–activity relationship studies of BAM15.

Using the BAM15 scaffold as a prototype, second generation analogs were developed (Figure 3).^47^ By substituting one of BAM15′s aniline groups for a hydroxy moiety, compounds with improved properties were discovered. Specifically, to optimize the pyrazine core, structural evolution to a pyridine core generated **SHM115**, which had greater distribution in white and brown adipose tissues and was efficacious for the treatment of Western diet induced obesity in mice.^48^ While maintaining the pyrazine core of BAM15 and incorporating a hydroxy group, lead compound **SHS4121705** was revealed with a half-life of 5.7 h in mice. **SHS4121705** was primarily localized in the liver and was efficacious in a STAM mouse model of MASH.^47^ However, the STAM model is a chemically induced process that does not accurately mimic human MASH pathology.^49,50^ Thus, in this study, a more clinically relevant MASH-mouse model was employed.

In a continued effort to optimize the BAM15 scaffold, a new generation of mitochondrial uncouplers is described. The key transformation is the transposition of the hydroxy group from the 5 to 7-position (Figure 3). We hypothesized that the pyridine core would mimic the lipophilicity of **SHM115** and changing the hydroxy position could lead to enhanced potency while maintaining desirable pharmacokinetic properties. In this report, we installed unique aniline, biphenyl aniline, and phenyl groups onto the scaffold, then investigated their activity *in vitro* and *in vivo*. Our studies identified **SHO1122147** as a potent uncoupler with an EC_50_ of 3.6 μM. Additionally, it acts as an antiobesity and anti-MASH agent as determined by a more clinically relevant model of MASH, Gubra-Amylin NASH (GAN).

## Results

**Scheme 1:**
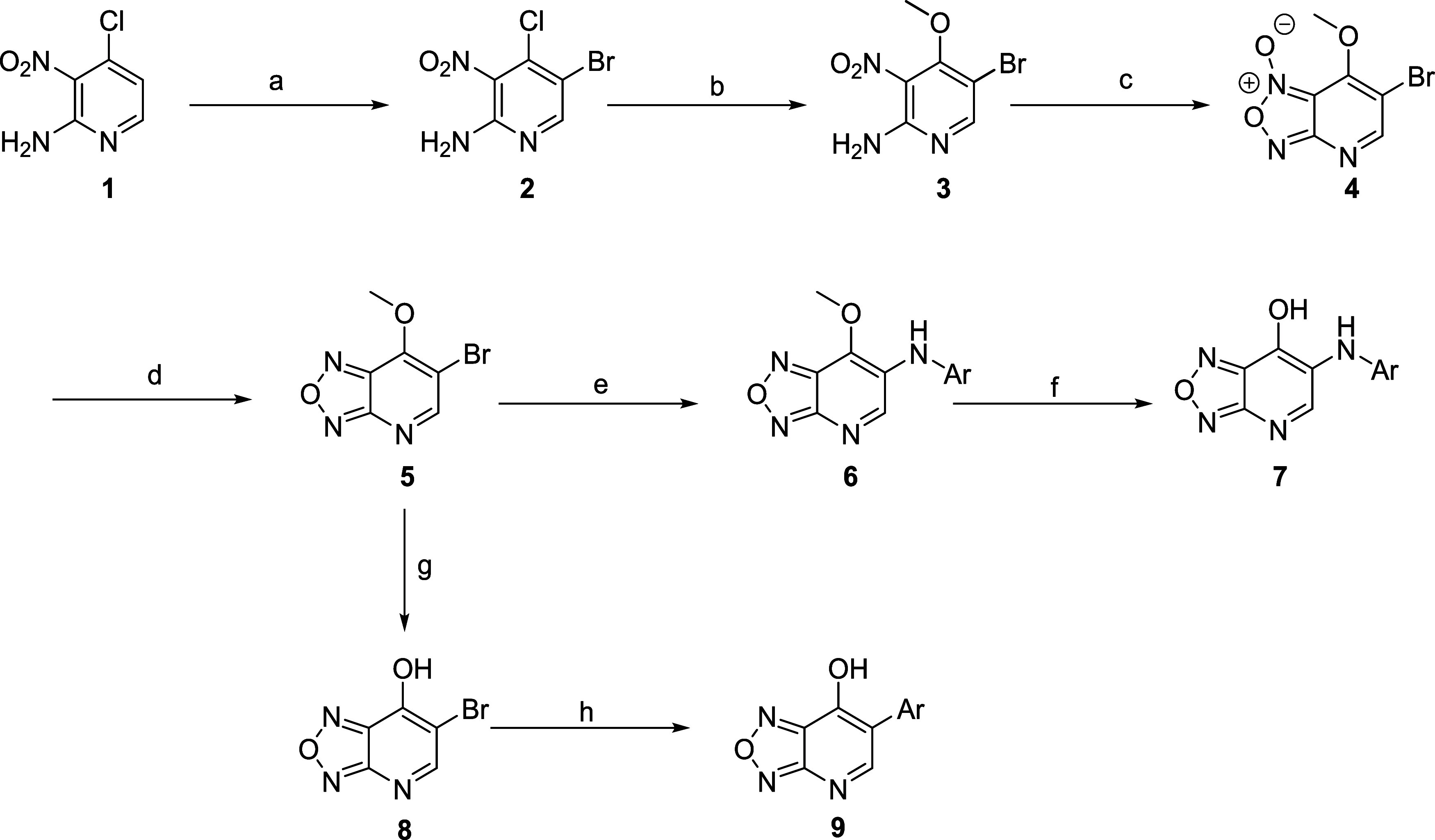
Synthetic Route for the 6-(Phenylamino)-[1,2,5]oxadiazolo[3,4-*b*]pyridin-7-ol Derivatives with Biphenyl Aniline and Phenyl Substitution in the 7-Position Reagents and conditions: (a) *N*-bromosuccinimide, acetonitrile, 80 °C, 1 h, 83%; (b) sodium methoxide, methanol, 35 °C, 16 h, 80%; (c) (diacetoxyiodo)benzene, acetonitrile, 80 °C, 1 h, 77%; (d) triphenylphosphine, CH_2_Cl_2_, 40 °C, 16 h, 75%; (e) Pd_2_(dba)_3_, Xantphos, K_2_CO_3_, aniline, toluene, 110 °C, 8 h; (f) Na_2_CO_3_, 1,4-dioxane, water (2:1), 110 °C, 4 h, 8–82% yield over two steps; (g) KOH, water, 1,4-dioxane, rt, 15 min; (h) Pd(dppf)Cl_2_·CH_2_Cl_2_, Ar–B(OH)_2_, Na_2_CO_3_, 1,4-dioxane, water, 90 °C, 18 h, 7–34% yield over two steps.

In this work, we aimed to investigate the synthetic route for the 6-(phenylamino)-[1,2,5]oxadiazolo[3,4-*b*]pyridin-7-ol derivatives with biphenyl aniline and phenyl substitution in the 7-position (Scheme 1). (**7**) began with commercially available 4-chloro-3-nitropyridin-2-amine **1**. Bromination with *N*-bromosuccinimide affords aniline **2** in good yield (83%). Nucleophilic aromatic substitution with sodium methoxide resulted in the formation of the 3-bromo-4-methoxy-substituted pyridine intermediate **3**, which was followed by cyclization to form the furoxan **4** using (diacetoxyiodo)benzene. Reduction of the furoxan ring by triphenylphosphine generated 6-bromo furazanopyridine intermediate **5**. Buchwald-Hartwig cross-coupling reactions between anilines, intermediate **5**, and subsequent deprotection with K_2_CO_3_ in dioxane/water afforded the desired product **7**, 7-hydroxyoxadiazolopyridine. Alternatively, phenyl substituted derivatives (**9**) were synthesized from 6-bromo-7-methoxy-[1,2,5]oxadiazolo[3,4-*b*]pyridine (**5**) by deprotecting the methoxy group with potassium hydroxide to yield **8**. A Pd-catalyzed Suzuki-Miyaura coupling reaction generated various phenyl derivatives (**9**).

With **7** and **9** derivatives in hand, we screened for mitochondrial uncoupling activity as a function of oxygen consumption rate (OCR). OCR increases when a mitochondrial uncoupler transports protons across the intermembrane of the mitochondria to the matrix, allowing the cell to increase respiration. Thus, increased respiration is detected by OCR. L6 rat myoblasts were treated with compounds for 90 min and subsequently analyzed on an Agilent Seahorse XF instrument (All compounds tested were >95% pure by UPLC analysis). Experiments were conducted at 8 different concentrations (0.37, 1.1, 3.3, 10, 25, 50, 100, and 200 μM) over the course of 200 min. Because OCR results are influenced by both cell density and quality, BAM15 was used as both as a positive control and standard for maximum OCR. OCR data was normalized by comparing area under the curve relative to BAM15. The minimum threshold for uncoupler properties includes an EC_50_ value <5 μM and an OCR value >35% of BAM15.

The structure–activity relationship study (SAR) began by investigating an aniline (**7a**) to determine base activity (Table 1). While active as an uncoupler, a significant drop in both efficacy and potency was observed (40%, 59 μM) when compared with BAM15. Further functionalization of the aniline allowed for tuning of the lipophilicity and acidity of the scaffold. Thus, an array of alkyl substituents in the *para* position (**7b**–**7e**) were synthesized and all but *t*-butyl aniline (**7e**) were tolerated. The most promising compound was the *para*-substituted ethyl tail **7c**, with 50% of BAM15 OCR and an EC_50_ of 14 μM. Unfortunately, alkyl-substituted analogs were found to have short half-life values in mice so further investigation of this series was not pursued (data not shown). An electron donating *t-*butyl ether analog (**7f**) had poor activity with only 20% BAM15. Several strong withdrawing groups (**7i**–**7j**) were then added in the *para* position. The only tolerated group was -SCF_3_ (**7h**) but again its activity was reduced. The 3-aminopyridine analogs **7k** and **7l** were also inactive.

**Table 1:**
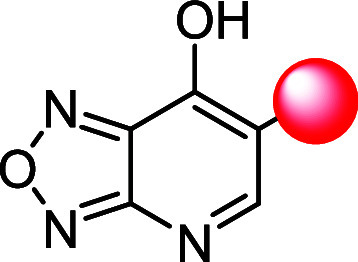

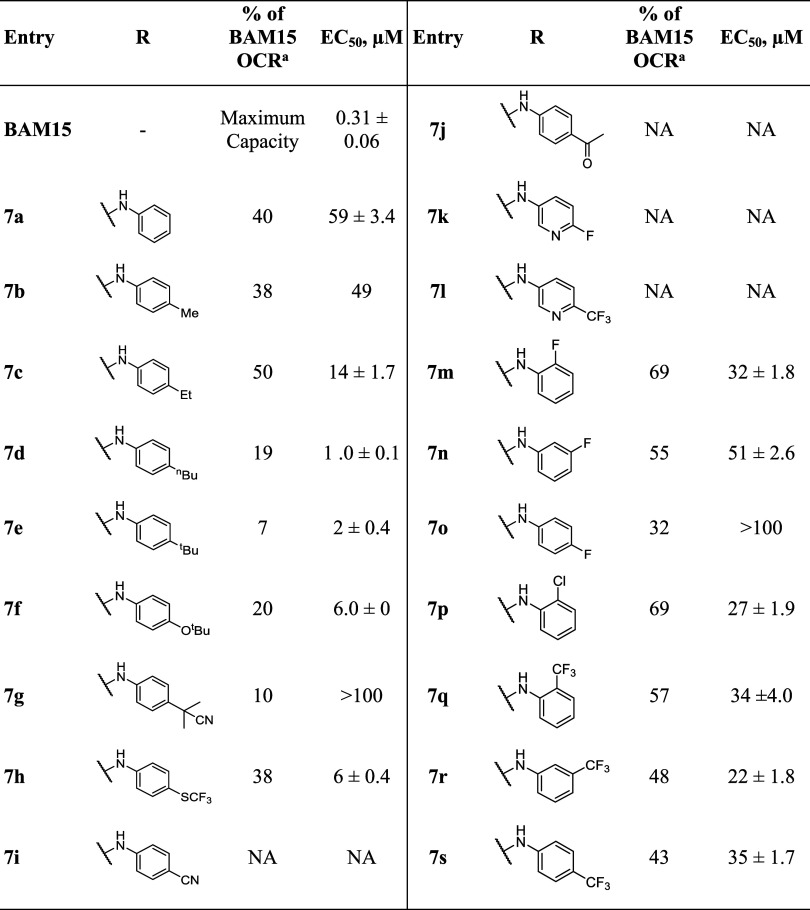
OCR Data of 6-Substituted Oxadiazolopyridine Derivatives

a Ratio of integrated area under OCR dose curve above baseline relative to that of BAM15 from the same experiment expressed as percent. NA = No Activity. Highest tested concentration is 200 μM. BAM15 was tested up to 200 μM.

Inspired by previous work, halogen substituents were added in the *ortho*, *meta*, and *para* positions to identify the most optimal position.^48^ The *ortho*-fluorinated **7m** exhibited 69% of BAM15 uncoupling capability, a 72% increase when compared with the nonsubstituted aniline. However, fluorine substitution in the *meta* (**7n**) and *para* positions (**7o**) both decreased the OCR activity (55 and 32%, respectively). Substitution of the *ortho*-fluoro group for a chloro group (**7p**) resulted in improved EC_50_ (27 μM), suggesting that the *ortho*-chloro group increases potency. Use of the strongly electron withdrawing trifluoromethyl group in the *ortho*, *meta*, and *para* positions (**7q**–**7s**) was equally effective.

With the promising results from the monosubstituted aniline derivatives, multisubstituted analogs were investigated with fluorine in the *ortho* position (Table 2). All the difluoro-substituted aniline derivatives (**7t**–**7w**) had OCR values of >50% that of BAM15. However, the EC_50_ values remained high (>40 μM). Additional fluorine atoms resulted in a successive improvement, but pentafluoroaniline **7z** was the most potent. The 2-fluoro-3-chloro analog **7aa** was also relatively potent at 32 μM. When a trifluoromethyl group was placed around the ring instead of chlorine in compounds **7ab**, **7ac**, and **7ad** the potency improved with derivative **7ac** having an EC_50_ of 11 μM. Other variations did not significantly improve activity (**7ae**–**7ag**).

**Table 2:**
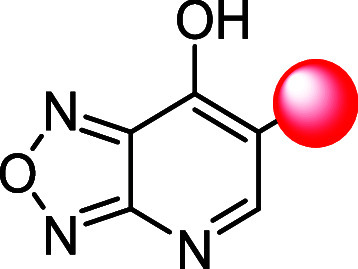

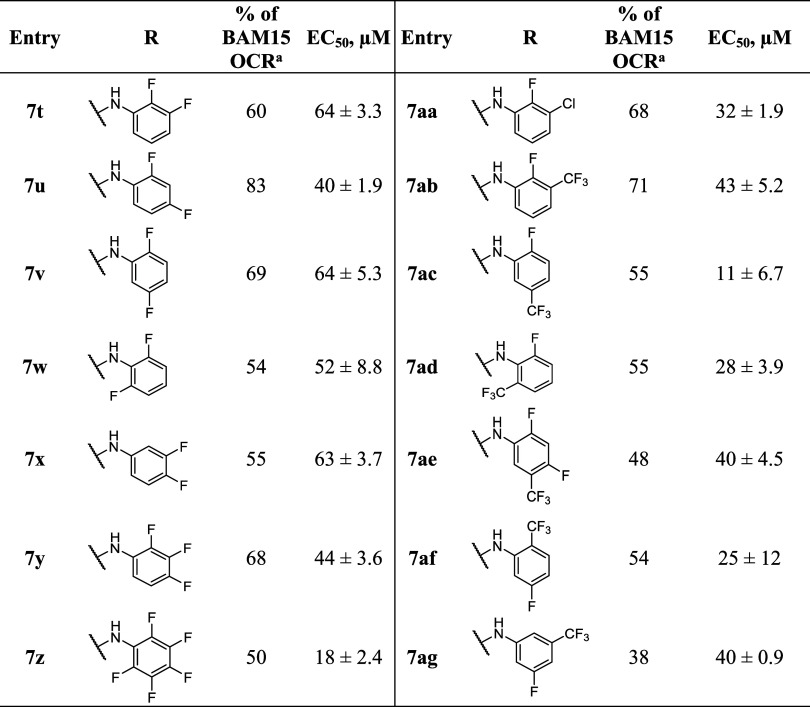
OCR Data of Multi-Substituted Aniline Derivatives

a Ratio of integrated area under OCR dose curve above baseline relative to that of BAM15 from the same experiment expressed as percent. NA = No Activity. Highest tested concentration is 200 μM. BAM15 was tested up to 200 μM.

We next investigated the effect of the addition of an aryl ring to the aniline moiety (Table 3). 2-Fluoro-4-phenyl aniline **7ah** showed limited OCR activity (10%). We then tested OCR for 2- and 3- phenyl anilines (**7ai**, **7aj**) but neither were tolerated. Unfortunately, we were unsuccessful at synthesizing the 4-phenyl aniline analog. Next, we investigated the effect of substitutions on the pendant ring. First, the 2′*-*fluoro analog **7ak** was not tolerated. This was followed by the *meta*-fluoro (**7al**), *meta-*chloro (**7am**), and *meta-*trifluoromethyl (**7n**) moieties. In this series we observe a significant improvement in both efficacy and potency, with **7al** and **7am** having EC_50_ values of 1.2 and 3.6 μM, respectively. It was noted that the (**7an**) showed cellular toxicity at higher concentrations. Interestingly, when fluorine (**7ao**) or chlorine (**7ap**) atoms were placed on the 4′ position, the activity of the compounds were not only in the low μM regime, but now possess favorable (<50%) OCR activity relative to BAM15. However, the 4′*-*trifluoromethoxy analog **7aq** exhibited toxicity and was not tolerated. Unfortunately, further structural changes with pyridyl trifluoromethyl groups or disubstitution with halogens (**7ar**–**7aw**) resulted in decreased activity. Likewise, introducing fused ring systems in **7ax** and **7ay** also did not improve activity.

**Table 3:**
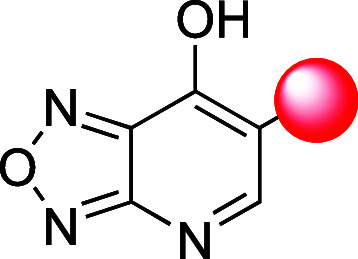

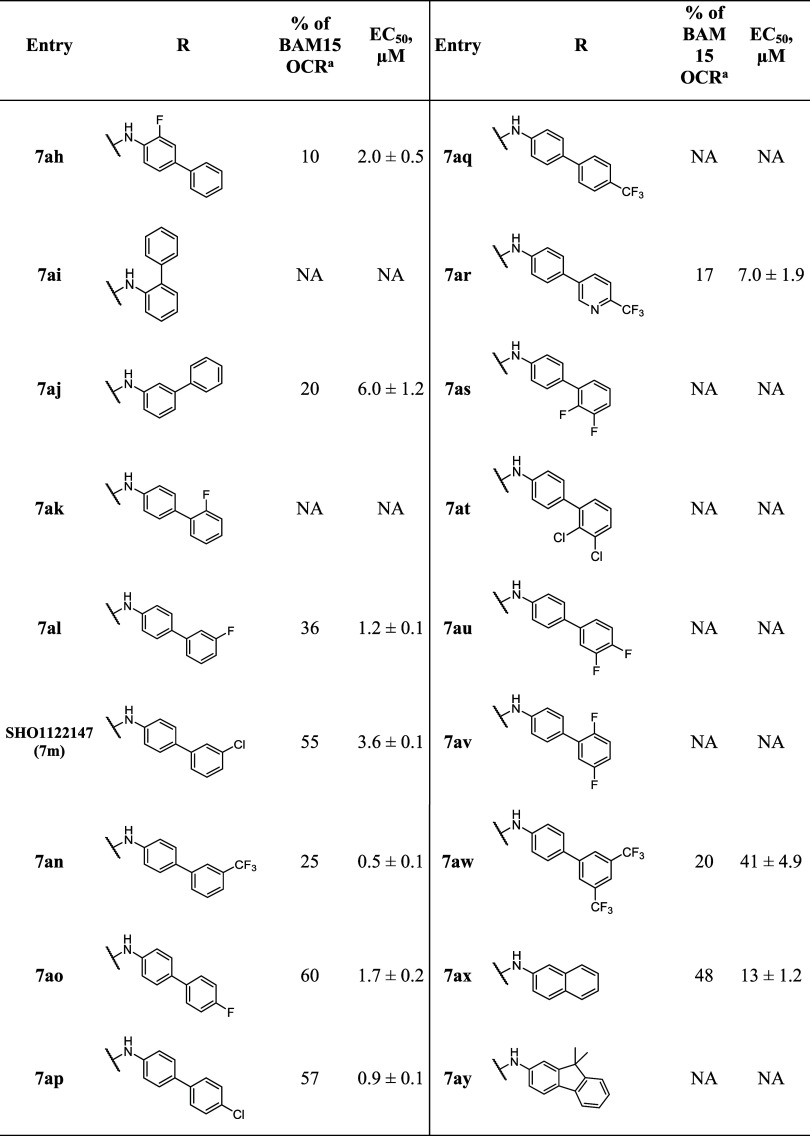
OCR Data of 4-[Phenyl]aniline Derivatives

a Ratio of integrated area under OCR dose curve above baseline relative to that of BAM15 from the same experiment expressed as percent. NA = No Activity. Highest tested concentration was 200 μM. BAM15 was tested up to 200 μM.

We next turned our attention to 6-phenyloxadiazolopyridine analogs where the aniline NH was removed. As shown in Table 4, 2-fluorophenyl analog (**9a**) had an EC_50_ of 27 μM and 69% BAM15 activity. The chlorinated surrogate (**9b**) was less potent but moving the chlorine atom to the *meta* (**9c**) and *para* (**9e**) positions resulted in progressive improvement in potency with compound **9e** having an EC_50_ of 12 μM and 52% BAM15 activity.

**Table 4:**
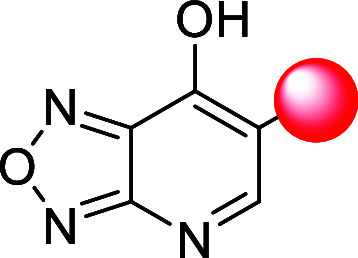

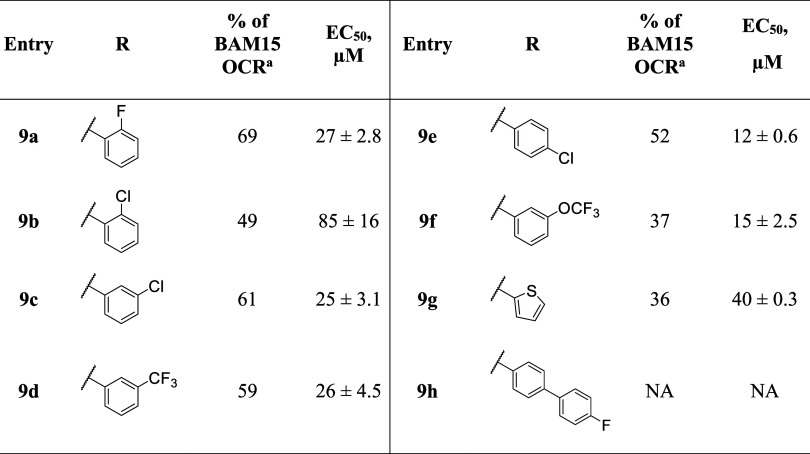
OCR Data of 6-Phenyl Oxadiazolopyridine Analogs

a Ratio of integrated area under OCR dose curve above baseline relative to that of BAM15 from the same experiment expressed as percent. NA = No Activity. Highest tested concentration is 200 μM. BAM15 was tested up to 200 μM.

Trifluoromethyl and trifluoromethoxy substituted derivatives **9d** and **9f**, while active, exhibited cellular toxicity at higher concentrations (data not shown). An analog with a heterocyclic group such as thiophene (**9g**) showed poor activity. Finally, a 4′-fluorophenyl analog (**9h**) which is the biphenyl analog of potent and efficacious **7ao**, was shown to be inactive. Overall, our studies suggest that the aniline NH was not necessary for uncoupling activity.

**Figure 4:**
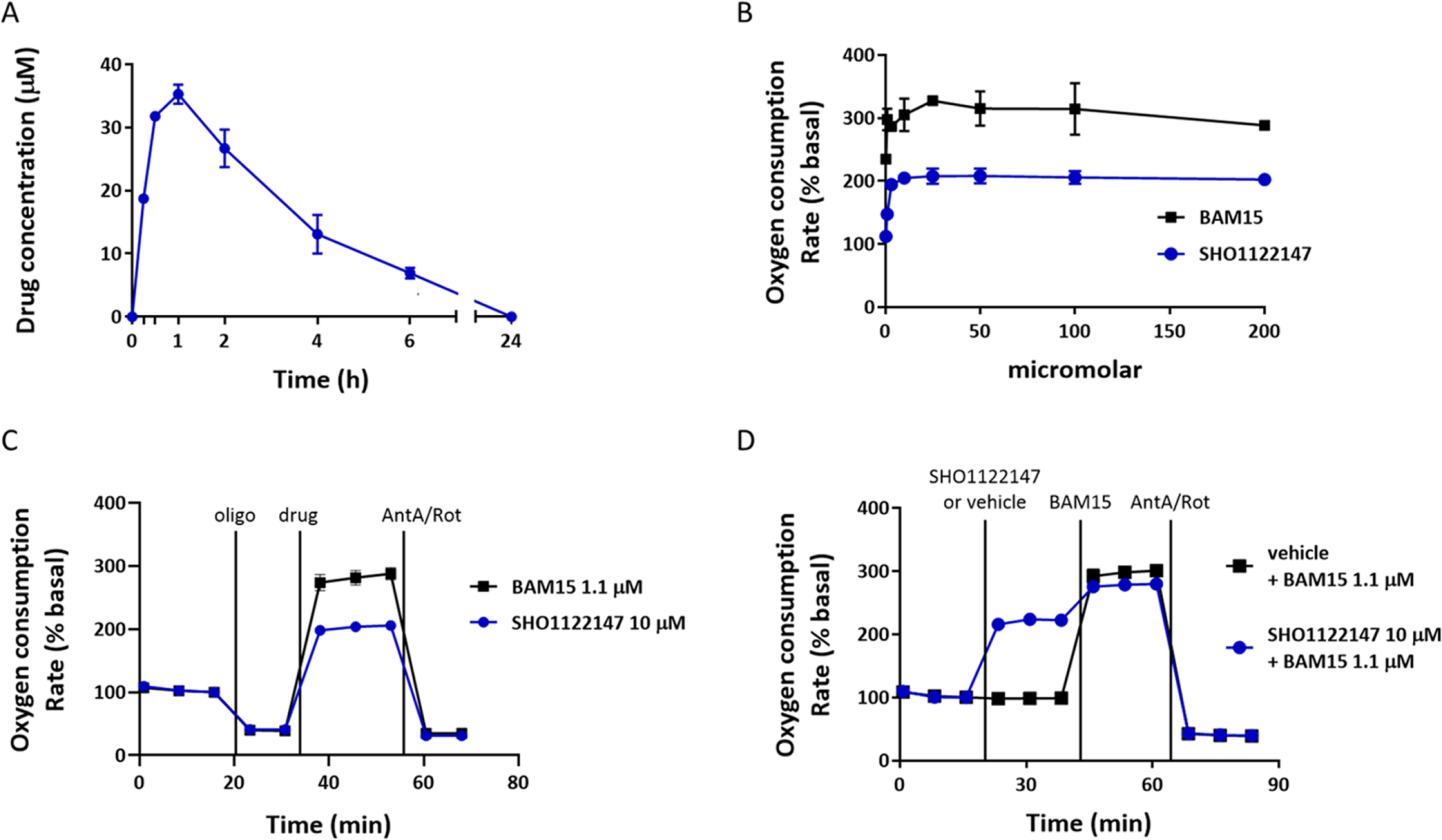
**SHO1122147** is an orally bioavailable protonophore. (A) Pharmacokinetic properties of **SHO1122147** administered to C57BL/6J male mice by oral gavage at 10 mg kg^–1^ (*n* = 3). (B) Dose–response curves of **SHO1122147** and BAM15-stimulated L6 myoblast cells. (C) Mitochondrial stress test in L6 myoblast cells dosed sequentially with oligomycin (oligo, 1 μM) or vehicle, **SHO1122147** (10 μM) or BAM15 (1.1 μM) with vehicle, and antimycin A (AntA, 10 μM) plus rotenone (rot, 1 μM) at the indicated time. *n* = 3 wells per condition. Values are represented as mean ± standard error of the mean (SEM) (D) Self-limiting test in L6 myoblast cells–dosed with **SHO1122147** (10 μM) followed by BAM15 (1.1 μM) and antimycin A (AntA, 10 μM) plus rotenone (Rot, 1 μM) were added at the indicated time. *n* = 4 wells per condition from three separate experiments.

To advance compounds toward an efficacy study in a mouse model of MASH, we set a threshold of >35% BAM15 OCR activity and <5 μM EC_50_ and performed pharmacokinetic studies in mice (Table 5). Mice were dosed with single bolus (10 mg kg^–1^) of **7ao**, **7ap**, **7al**, and **7m**. The exposure levels in plasma were determined by liquid chromatography/mass spectrometry (LC/MS) and half-life was calculated. While compounds **7ao**, **7ap**, and **7al** had low exposure levels (4–6 μM), **7m** (**SHO1122147**) had suitable exposure (*C*_max_ = 35 μM) and half-life of 2.0 h (Figure 4A). With a candidate compound selected, we performed a mitochondrial stress test to determine whether **SHO1122147** has protonophore mechanism of action. As expected, the OCR activity of BAM15 was more potent than **SHO1122147** (Figure 4B). Blockade of respiration with oligomycin, an ATP synthase inhibitor, at 20 min decreased respiration as judged by the decrease in OCR (Figure 4C). This is expected as protons no longer reenter the mitochondrial matrix via ATP synthase. However, the addition of **SHO1122147** or BAM15 (*t* = 40 min) increased OCR suggesting proton transport into the matrix independent of ATP synthase. **SHO1122147**′s OCR activity is lower than the maximum rate for BAM15, and maximum OCR rate is consistent (compare Figure 4B–C). The OCR activity in the presence of oligomycin continued until the addition of complex I and complex III inhibitors (antimycin A and rotenone) where a drop in OCR was observed. These results indicate that the activity of **SHO1122147** requires mitochondrial electron transport chain activity. Taken together, our studies indicate that the OCR observed occurs through the mitochondrial electron transport chain with mitochondrial uncoupling activity. Additionally, **SHO1122147** was tested in conjunction with BAM15 to determine whether maximum respiration could be exceeded. As shown in Figure 4D, **SHO1122147** alone increases respiration to 200% but upon the addition of BAM15 reaches the maximum of 300%, the same result as when cells are treated with only BAM15. These results suggest that **SHO1122147** acts in a self-limiting manner and does not inhibit the maximum mitochondrial respiration.

**Table 5:**
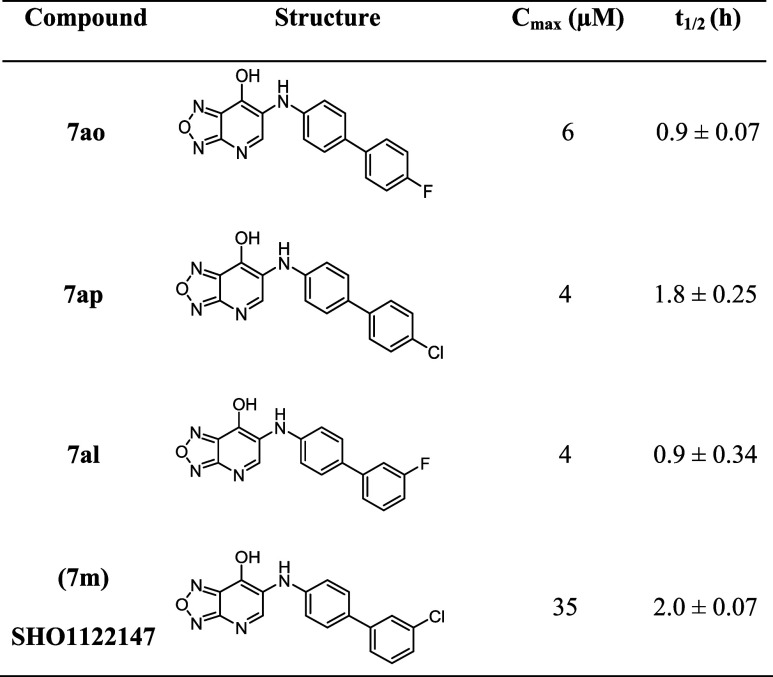
Pharmacokinetic Properties of Select Analogs in Mice

a Compounds were administered at 10 mg kg^–1^ of body weight by oral gavage to C57BL/6 male mice. Plasma samples were collected over 24 h after gavage and analyzed by LC-MS/MS. *C*_max_ = maximum plasma concentration. *t*_1/2_ = half-life.

**Figure 5:**
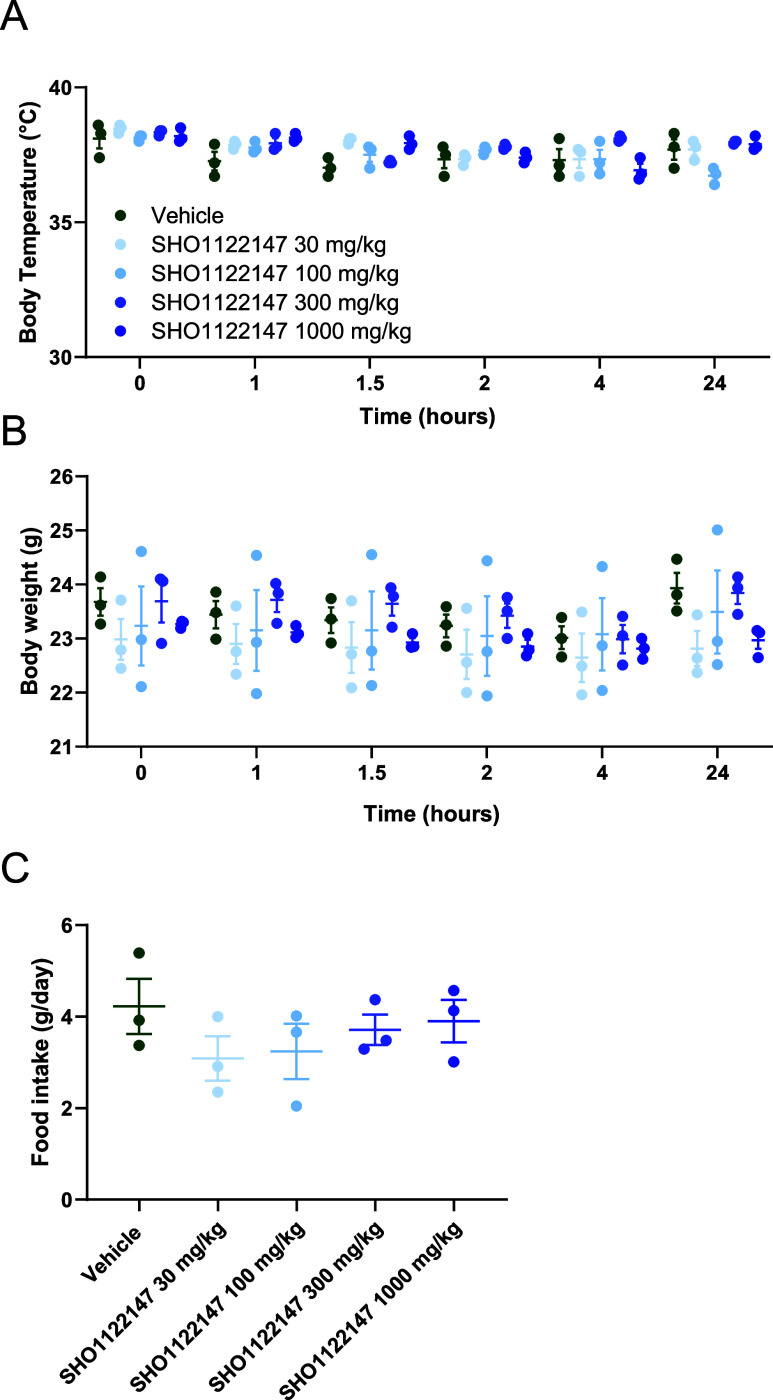
No observed adverse effect level (NOAEL) study with **SHO1122147**. (A) Body temperature, (B) body weight, and (C) average food intake measurements following administration of vehicle or **SHO1122147** at the indicated doses. Values are represented as mean ± SEM (*n* = 3).

We next assessed **SHO1122147** tolerability in mice by conducting a no observed adverse effect level (NOAEL) study. Rectal temperature, food intake, and body weight were monitored over the course of 24 h after oral dosing with vehicle or **SHO1122147** (30, 100, 300, and 1000 mg kg^–1^) (Figure 5). We observed no changes in temperature, weight, or appetite for all **SHO1122147** doses when compared to the vehicle group. These results indicate that the compound was well tolerated acutely at doses up to 1000 mg kg^–1^.

Finally, we assessed **SHO1122147** efficacy in a mouse model of MASH. We utilized the diet induced Gubra-Amylin NASH (GAN) because it is a more clinically relevant model than the chemically induced STAM model used in our past work. Thus, C57BL/6 male mice were fed GAN diet for 33 weeks before the GAN diet was supplemented with or without **SHO1122147** at 200 mg kg^–1^ admixed in food. **SHO1122147** was admixed in food due to its 2-h half-life and the 200 mg kg^–1^ dose was chosen because it was at least 5-fold lower than the maximum tolerated dose. Food intake was monitored daily during treatment and no significant changes in caloric intake were observed between groups.

**Figure 6:**
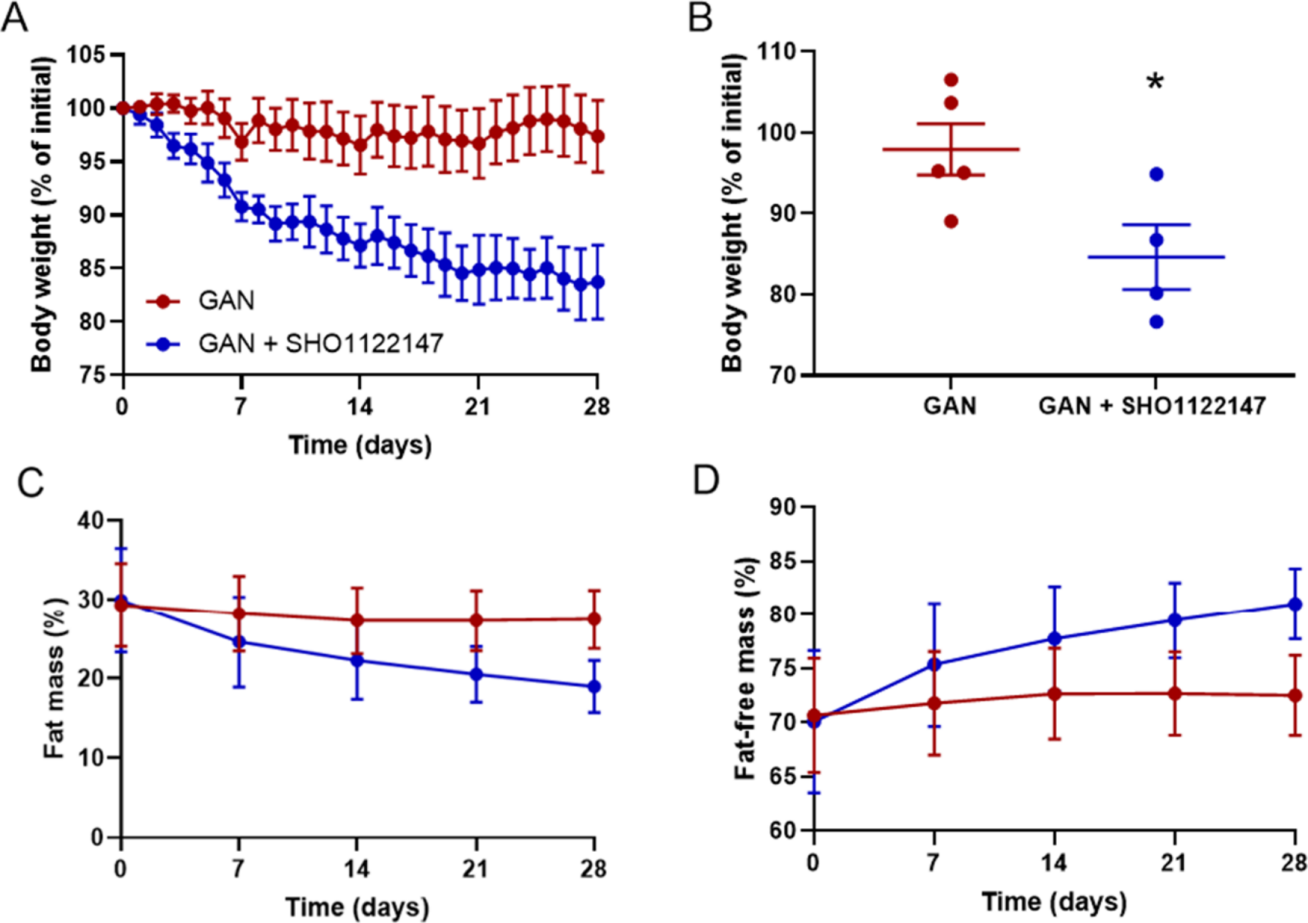
**SHO1122147** decreases body weight while improving body composition in the GAN mouse model of NASH. (A, B) Body weight represented as percentage of initial weight (A) over time and (B) at the end of treatment. Measurements of (C) body fat and (D) lean mass represented as percentage of body weight over time. Values are shown as mean ± SEM (*n* = 4–5). *p < 0.05 compared with GAN, determined by unpaired parametric *t* test (two-tailed).

**Figure 7:**
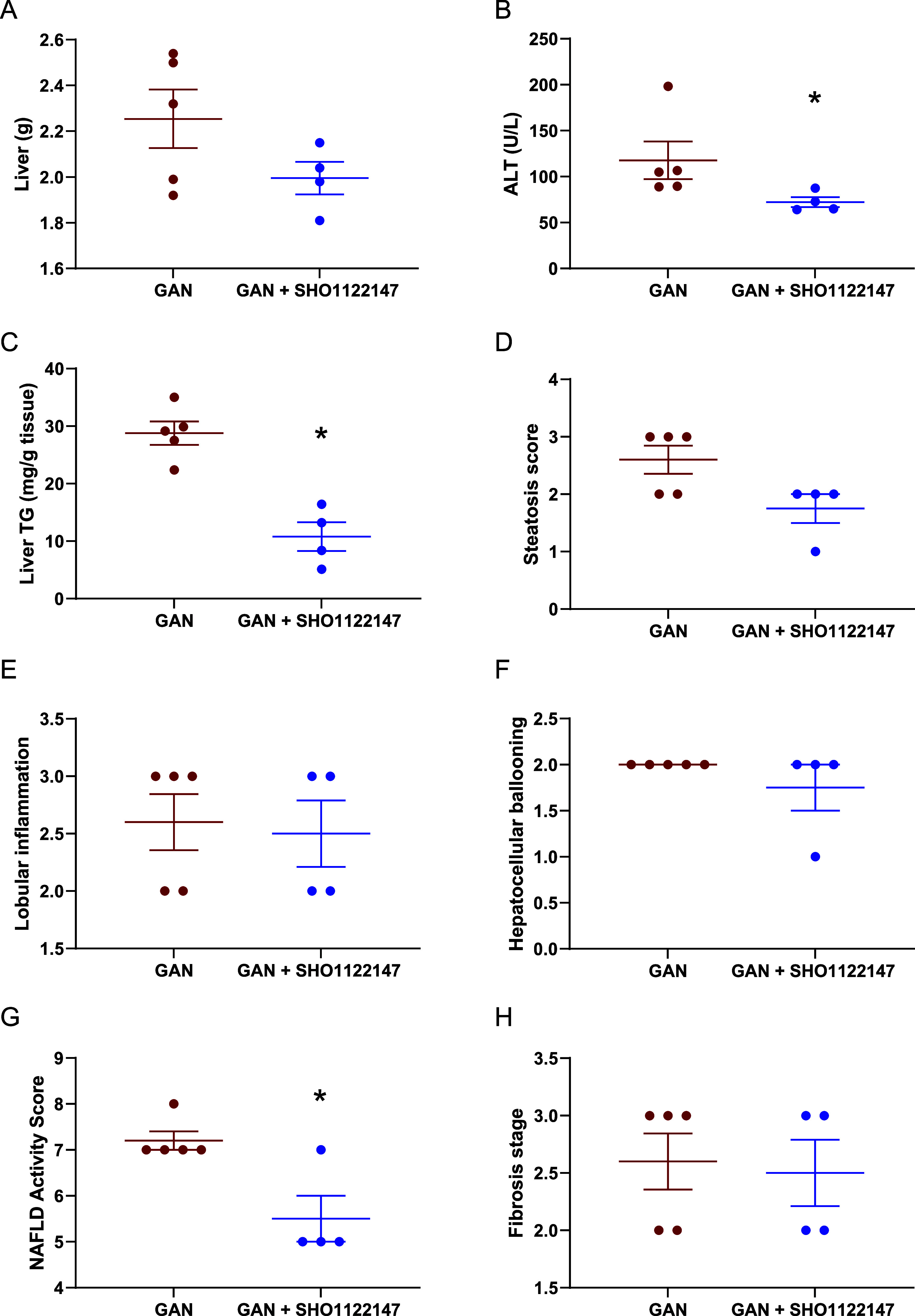
**SHO1122147** decreases liver steatosis and lowers ALT in a mouse model of NASH. (A) Liver weight and (B) plasma alanine aminotransferase (ALT) activity levels. (C) Liver triglycerides (TG) at the end of the study. (D–F) Individual and (G) composite NAFLD activity scores. (H) Liver fibrosis stage. Values are represented as mean ± SEM (*n* = 4–5). *p < 0.05 compared with GAN, determined by (C) unpaired parametric *t* test (two-tailed) and (B, G) Mann–Whitney nonparametric test.

Following 4 weeks of compound treatment, we observed that mice fed **SHO1122147** had lost 15.4% of body weight (Figure 6A,B). Body composition measured weekly by EchoMRI analysis showed that **SHO1122147** treatment improved body composition by lowering fat mass and increasing fat-free lean mass as a percent of body weight (Figure 6C,D). At end of in-life studies, terminal blood was collected and organ weights recorded. Mice treated with **SHO1122147** had no significant change in liver mass but showed improvements in fatty liver disease phenotypes including decreased plasma alanine aminotransferase (ALT) and decreased liver triglycerides (Figure 7A–C). Hepatopathological assessment of fixed liver samples revealed a significant decrease of the MAFLD activity score that was primarily due to decreased hepatic steatosis (Figured 7D–G). No changes were observed in the fibrosis stage of **SHO1122147** liver samples as compared to GAN controls. These results suggest that mitochondrial uncouplers have the potential to decrease bodyweight and improve MASH score driven by hepatic steatosis.

## Conclusions

There is significant interest in using small molecule mitochondrial uncouplers for the treatment of obesity and accompanying metabolic disorders, such as MASH, due to the novel approach of increasing energy consumption versus reducing energy intake. Herein, we performed a SAR of a new mitochondrial uncoupler scaffold, [1,2,5]oxadiazolo[3,4-*b*]pyridin-7-ol, where aniline or phenyl derivatives were placed in the 6-position. Our SAR revealed electron withdrawing groups, such as halogens and trifluoromethyl groups on the aniline moiety, afforded mitochondrial uncouplers with moderate EC_50_ values and % BAM15 OCR. In general, the 4-phenylaniline series were better than the aniline and phenyl derivatives, with analogs bearing fluorine or chlorine on the *ortho-* or *para-* positions preferred. Characterization of **SHO1122147** confirmed that it is a *bona fide* mitochondrial uncoupler. Efficacy studies in the GAN mouse model demonstrated a decrease in weight, liver fat, and MASH activity score. The ALT and liver triglyceride levels also significantly improved. Overall, these studies suggest the potential of mitochondrial uncouplers as therapeutics for metabolic diseases. Further studies are ongoing to improve the potency and pharmacokinetic profile of the structure, which will be disclosed in due course.

## Experimental Section

### General Material and Synthetic Procedures

Reagents were obtained from commercial sources unless stated otherwise. Dioxane was deoxygenated by bubbling in argon for 30 min. Toluene was dried using the Innovative Technology Pure SolvMD solvent purification system. Flash silica gel chromatography was performed using SiliaFlash P60 40–63 μm, 60 Å. Thin-layer chromatography was performed to determine the reaction progress utilizing Silicycle aluminum backed silica gel F-254 plates. An Agilent 400-MR 400 MHz or a Varian Inova 400 MHz were used for ^1^H and ^19^F NMR spectroscopic experiments. A Bruker Avance II 500 MHz was predominately utilized for ^13^C NMR spectroscopic experiments unless stated otherwise. ^1^H and ^13^C NMR spectra are referenced to an internal standard (acetone-*d*_6_), and all chemical shifts are reported in δ ppm. NMR spectra characterizations are presented as follows: chemical shift, multiplicity (s = singlet, d = doublet, t = triplet, q = quartet, dd = doublet of doublets, dt = doublet of triplets, dq = doublet of quartets, tt = triplet of triplets, qd = quartet of doublets, and m = multiplet), coupling constants (Hz), and integration. Purity assessment of compound was performed by Waters UPLC analysis. UPLC conditions: Purity assessments were performed by Waters UPLC analysis. UPLC conditions method one: Solvent A: Water (0.1% TFA); solvent B: acetonitrile (0.1% TFA); column: Acquity BEH C18 1.7 μm 2.1 mm × 50 mm; method: isocratic 95% A, 5% B from 0 to 3.40 min then linear gradient from 5 to 95% B by 5.10 min, return to 5% B by 5.95 min, then hold for 2 min at 95% A, 5% B; UV wavelength = 280 nm; flow rate: 0.613 mL/min. Method two: Solvent A: Water (0.1% TFA); solvent B: acetonitrile (0.1% TFA); column: Acquity BEH C18 1.7 μm 2.1 mm × 50 mm; method: isocratic 60% A, 40% B from 0 to 3.50 min then linear gradient from 40 to 95% B by 5 min, return to 40% B by 6 min, then hold for 2 min at 60% A, 40% B; UV wavelength = 280 nm; flow rate: 0.613 mL/min. All final materials synthesized had a purity ≥95% as measured by UPLC unless otherwise noted. All compounds tested *in vitro* and *in vivo* are >95 and >99% pure by HPLC, respectively.

#### 5-Bromo-4-chloro-3-nitropyridin-2-amine (**2**)

To a stirring solution of 4-chloro-3-nitropyridin-2-amine 1 (20.0 g, 115 mmol) in acetonitrile (500 mL) was added *N*-bromosuccinimide (24.6 g, 138 mmol). The reaction was heated under reflux for 1.5 h. The reaction mixture was allowed to cool to 40 °C and precipitated in cold water (500 mL). The precipitate was filtered, washed twice with water and dried to afford 2 (24.2 g, 95.9 mmol, 83%) as a brown, amorphous solid: ^1^H NMR (500 MHz, acetone-*d*_6_) δ 8.43 (s, 1H). ^13^C NMR (126 MHz, acetone-*d*_6_) δ 153.66, 152.70, 137.97, 107.82. HRMS: (ESI) [M – H]^+^ calcd for C_5_H_3_BrClN_3_O_2_, 251.9170, observed 251.9170 *m*/*z*. Δ 0.0 ppm.

#### 5-Bromo-4-methoxy-3-nitropyridin-2-amine (**3**)

A mixture of 5-bromo-4-chloro-3-nitropyridin-2-amine (**2**) (47.2 g, 187 mmol) and 25% sodium methoxide in methanol (72.8 g, 337 mmol) in methanol (500 mL) was stirred at 40 °C for 16 h. The reaction mixture was allowed to cool to room temperature and precipitated in cold water (500 mL). The precipitate was filtered, washed twice with water, and dried to afford **3** (37.2 g, 80%) as a yellow, amorphous solid: ^1^H NMR (400 MHz, acetone-*d*_6_) δ 8.29 (s, 1H), 6.74 (s, 2H), 4.03 (s, 3H). ^13^C NMR (126 MHz, acetone-*d*_6_) δ 159.75, 155.62, 154.29, 127.65, 101.69, 62.84. HRMS: (ESI) [M + H]^+^ calcd for C_6_H_6_BrN_3_O_3_, 247.9665, observed 247.9664 *m*/*z*. Δ −0.4032 ppm.

#### 6-Bromo-7-methoxy-1l4-[1,2,5]oxadiazolo[4,3-*b*]pyridin-1-olate (**4**)

5-Bromo-4-methoxy-3-nitropyridin-2-amine **3** (17.0 g, 69.0 mmol) and iodobenzene diacetate (61.8 g, 192 mmol) were added to a round-bottom and stirred in acetonitrile (400 mL) at 80 °C for 1 h. The reaction was then cooled to room temperature and poured into cold water (500 mL). The acetic acid was quenched with sodium bicarbonate until pH = 7. The reaction mixture was filtered, and the filtrate was extracted with ethyl acetate (4 times). The organic extracts were combined, dried over anhydrous sodium sulfate, concentrated *in vacuo*, and purified via chromatography on SiO_2_ (gradient: 0–15% ethyl acetate/hexanes) to afford **4** (13.1 g, 53 mmol, 77%) as a yellow, amorphous solid: ^1^H NMR (500 MHz, acetone-*d*_6_) δ 8.58 (s, 1H), 4.69 (s, 3H). ^13^C NMR (126 MHz, acetone-*d*_6_) δ 158.88, 154.09, 145.44, 127.69, 106.88, 64.04. HRMS: (ESI) [M + H]^+^ calcd for C_6_H_4_BrN_3_O_3_, 245.9509, observed 245.9512 *m*/*z*. Δ 1.2197 ppm.

#### 6-Bromo-7-methoxy-[1,2,5]oxadiazolo[3,4-*b*]pyridine (**5**)

To a stirring solution of, 6-bromo-7-methoxy-1l4-[1,2,5]oxadiazolo[4,3-*b*]pyridin-1-olate **4** (13.0 g, 52.0 mmol) was dissolved in dichloromethane (250 mL) and triphenylphosphine (15.2 g, 58.0 mmol) was added slowly at 0 °C. The mixture was allowed to warm to room temperature and stirred under reflux at 40 °C for 16 h. The reaction was concentrated *in vacuo*, and purified via chromatography on SiO_2_ (gradient: 0–10% ethyl acetate/hexanes) to afford **5** (9.1 g, 40 mmol, 75%) as an off-white, amorphous solid: ^1^H NMR (400 MHz, acetone-*d*_6_) δ 8.91 (s, 1H), 4.69 (s, 4H). ^13^C NMR (101 MHz, acetone-*d*_6_) δ 163.61, 161.58, 153.50, 141.40, 105.36, 62.84. HRMS: (ESI) [M + H]^+^ calcd for C_6_H_4_BrN_3_O_2_, 229.95597, observed 229.9561 *m*/*z*. Δ 0.8697 ppm.

##### General Procedure for e

To a sealed vial containing Pd_2_dba_3_ (0.1 equiv), Xantphos (0.2 equiv), 6-bromo-7-methoxy-[1,2,5]oxadiazolo[3,4-*b*]pyridine (**5**) (1.00 equiv), and K_2_CO_3_ (2.5 equiv) in toluene (0.2 M) was added the aniline derivative (1.1 equiv) under a nitrogen atmosphere with stirring. The reaction mixture was heated under reflux for 14 h. The mixture was allowed to cool to room temperature, concentrated *in vacuo*, and purified via chromatography on SiO_2_ (solvent system: ethyl acetate/hexanes) to afford a crude mixture of products **6** and residual aniline.

##### General Procedure for f

Methoxy derivatives **6** (1 equiv) and Na_2_CO_3_ (3 equiv) were suspended in dioxane (0.5 mL) and water (0.5 mL). The reaction was heated under reflux for 4 h, then allowed to cool to room temperature, diluted with water, and acidified with 10% aq. HCl. The resulting precipitate was filtered, washed with water, dried, and purified via chromatography on SiO_2_ (solvent system: ethyl acetate/hexanes) to afford the desired products **7**. Yields are listed as combined over two steps.

#### 6-(Phenylamino)-[1,2,5]oxadiazolo[3,4-*b*]pyridin-7-ol (**7a**)

Red solid (19%, 13 mg) ^1^H NMR (400 MHz, acetone-*d*_6_) 11.23 (s, 1H), 8.17 (s, 1H), 7.23–7.14 (m, 2H), 7.06–6.97 (m, 2H), 6.79 (tt, *J* = 7.4, 1.1 Hz, 1H), 6.53 (s, 1H). ^13^C NMR (126 MHz, acetone-*d*_6_) δ 168.9, 152.8, 146.4, 145.2, 134.5, 129.9, 126.7, 120.1, 116.7. HRMS: (ESI) [M + H]^+^ calcd for C_11_H_8_N_4_O_2_, 229.0720, observed 229.0726 *m*/*z*. Δ 2.6192 ppm.

#### 6-(*p*-Tolylamino)-[1,2,5]oxadiazolo[3,4-*b*]pyridin-7-ol (**7b**)

Purple solid (8.34%, 10 mg) ^1^H NMR (500 MHz, acetone-*d*_6_) δ 8.11 (s), 7.03 (d, *J* = 8.4 Hz, 2H), 6.96 (d, *J* = 8.4 Hz, 2H), 6.43 (s), 2.23 (s). ^13^C NMR (500 MHz, acetone-*d*_6_) δ 151.9, 144.0, 142.5, 131.6, 129.5, 128.9, 126.9, 116.5, 116.4, 19.68. HRMS: ESI [M + H]^+^ calc for C_12_H_10_N_4_O_2_, 243.0877, observed, 243.0879. *m*/*z*. Δ 0.8227 ppm.

#### 6-((4-Ethylphenyl)amino)-[1,2,5]oxadiazolo[3,4-*b*]pyridin-7-ol (**7c**)

Red solid (18%, 31 mg) ^1^H NMR (500 MHz, acetone-*d*_6_) δ 11.14 (s, 1H), 8.12 (s, 1H), 7.07 (d, *J* = 8.5 Hz, 2H), 6.99 (d, *J* = 8.5 Hz, 2H), 6.44 (s, 2H), 2.54 (q, *J* = 7.6 Hz, 2H), 1.17 (t, *J* = 7.6 Hz, 3H). ^13^C NMR (126 MHz, acetone-*d*_6_) δ 151.8, 144.0, 142.7, 135.7, 128.4, 126.9, 119.7, 116.5, 27.8, 15.5. HRMS: (ESI) [M – H]^−^ calcd for C_13_H_12_N_4_O_2_, 255.08875, observed 255.0891 *m*/*z*. Δ 1.3720 ppm.

#### 6-((4-Butylphenyl)amino)-[1,2,5]oxadiazolo[3,4-*b*]pyridin-7-ol (**7d**)

Purple solid (33%, 47 mg) ^1^H NMR (600 MHz, acetone-*d*_6_) δ 11.14 (s, 1H), 8.12 (s, 4H), 7.05 (d, *J* = 8.7 Hz, 9H), 6.98 (d, *J* = 2.3 Hz, 9H), 6.43 (s, 3H), 2.52 (t, *J* = 7.7 Hz, 9H), 1.61–1.49 (m, 10H), 1.34 (h, *J* = 15.1 Hz, 10H), 0.91 (t, *J* = 7.8 Hz, 14H). ^13^C NMR (126 MHz, acetone-*d*_6_) δ 168.5, 152.6, 145.0, 143.6, 135.1, 132.0, 129.9, 127.9, 117.3, 35.4, 34.8, 22.9, 14.2. HRMS: ESI [M + H]^+^ calc for C_15_H_13_N_5_O_2_, 285.1346, observed, 285.1355. *m*/*z*. Δ 3.1564 ppm.

#### 6-((4-(*tert*-Butyl)phenyl)amino)-[1,2,5]oxadiazolo[3,4-*b*]pyridin-7-ol (**7e**)

Purple solid (29%, 28 mg) ^1^H NMR (600 MHz, acetone-*d*_6_) δ 8.14 (s, 1H), 7.25 (d, *J* = 2.9, 2.2 Hz, 2H), 6.98 (dd, *J* = 2.9, 2.2 Hz, 2H), 6.45 (s, 1H), 1.27 (s, 9H). ^13^C NMR (126 MHz, acetone-*d*_6_) δ 168.2, 152.7, 144.9, 143.3, 143.3, 132.7, 127.7, 126.7, 119.9, 116.9, 34.5, 31.8. HRMS: ESI [M + H]^+^ calc for C_15_H_16_N_4_O_2_, 285.1346, observed, 285.1353 *m*/*z*. Δ 2.4550 ppm.

#### 6-((4-Butoxyphenyl)amino)-[1,2,5]oxadiazolo[3,4-*b*]pyridin-7-ol (**7f**)

Purple solid (61%, 96 mg) ^1^H NMR (600 MHz, acetone-*d*_6_) δ 11.05 (s, 1H), 7.09 (d, *J* = 9.0, 5.8, 3.6 Hz, 2H), 6.87 (d, *J* = 8.9, 5.8, 3.6 Hz, 2H), 6.33 (s, 1H), 3.96 (t, *J* = 6.4 Hz, 2H), 1.74 (m, 2H), 1.50 (h, *J* = 15.0, 7.5 Hz, 2H), 0.99 (t, *J* = 7.4 Hz, 3H). ^13^C NMR (126 MHz, acetone-*d*_6_) δ 154.1, 151.6, 143.7, 137.3, 128.8, 127.9, 120.0, 119.6, 115.2, 67.6, 31.3, 19.0, 13.2. HRMS: ESI [M + H]^+^ calc for C_15_H_16_N_4_O_3_, 301.1295, observed, 301.1298. *m*/*z*. Δ 0.9962 ppm.

#### 2-(4-((7-Hydroxy-[1,2,5]oxadiazolo[3,4-*b*]pyridin-6-yl)amino)phenyl)-2-methylpropanenitrile (**7g**)

Purple solid (82%, 43 mg) ^1^H NMR (600 MHz, acetone-*d*_6_) δ 8.19 (s), 7.31 (d, *J* = 8.5, 5.1, 3.0 Hz, 2H), 6.99 (d, *J* = 8.5, 5.1, 3.0 Hz, 2H), 6.68 (s), 1.66 (s, 6H). ^13^C NMR (600 MHz, acetone-*d*_6_) δ 168.12, 152.1, 145.5, 144.6, 135.7, 132.2, 125.9, 125.1, 124.7, 115.2, 36.2, 28.4. HRMS: ESI [M + H]^+^ calc for C_15_H_13_N_5_O_2_, 296.1142, observed, 296.1147. *m*/*z*. Δ 1.6885 ppm.

#### 6-((4-((Trifluoromethyl)thio)phenyl)amino)-[1,2,5]oxadiazolo[3,4-*b*]pyridin-7-ol (**7h**)

Red solid (65%, 187 mg) ^1^H NMR (400 MHz, acetone-*d*_6_) δ 11.50 (s, 1H), 8.29 (d, *J* = 2.2 Hz, 1H), 7.43 (dd, *J* = 4.8, 2.4 Hz, 2H), 7.11 (s, 1H), 6.98 (d, *J* = 10.1 Hz, 2H). ^19^F NMR (376 MHz, acetone) δ −45.5. ^13^C NMR (126 MHz, acetone-*d*_6_) δ 169.6, 153.3, 150.9, 146.0, 140.5, 138.8, 130.9 (q, *J* = 307.1 Hz), 124.1, 115.7, 110.8. HRMS: (ESI) [M + H]^+^ calcd for C_12_H_7_F_3_N_4_O_2_S 329.0314 observed 329.0317 *m*/*z*. Δ 0.9117 ppm.

#### 4-((7-Hydroxy-[1,2,5]oxadiazolo[3,4-*b*]pyridin-6-yl)amino)benzonitrile (**7i**)

Red solid (23%, 52 mg) ^1^H NMR (500 MHz, acetone-*d*_6_) δ 11.56 (s, 1H), 8.29 (s, 1H), 7.47 (d, *J* = 8.7 Hz, 2H), 7.31 (s, 1H), 6.96 (d, *J* = 8.8 Hz, 2H). ^13^C NMR (126 MHz, acetone-*d*_6_) δ 153.4, 152.0, 146.1, 141.3, 134.1, 123.4, 120.5, 114.9, 100.8. HRMS: (ESI) [M – H]^−^ calcd for C_12_H_7_N_5_O_2_ 252.0527 observed 252.0517 *m*/*z*. Δ −3.9674 ppm.

#### 1-(4-((7-Hydroxy-[1,2,5]oxadiazolo[3,4-*b*]pyridin-6-yl)amino)phenyl)ethan-1-one (**17j**)

Yellow solid (62%, 88 mg) ^1^H NMR (400 MHz, acetone-*d*_6_) δ 8.16 (s, 1H), 7.83 (d, *J* = 8.8 Hz, 2H), 6.77 (d, *J* = 8.7 Hz, 2H), 2.49 (s, 3H). ^13^C NMR (126 MHz, MeOD) δ 153.4, 131.9, 128.4, 123.5, 114.0, 26.1. HRMS: (ESI) [M – H]^−^ calcd for C_13_H_10_N_4_O_3_, 269.0680, observed 269.0677 *m*/*z*. Δ −1.1149 ppm *Not all carbon peaks observed.

#### 6-((6-Fluoropyridin-3-yl)amino)-[1,2,5]oxadiazolo[3,4-*b*]pyridin-7-ol (**7k**)

Red solid (31%, 60 mg) ^1^H NMR (500 MHz, acetone-*d*_6_) δ 11.38 (s, 1H), 8.22 (s, 1H), 7.85 (t, *J* = 2.6 Hz, 1H), 7.50 (ddd, *J* = 8.7, 6.9, 3.1 Hz, 1H), 6.87 (dd, *J* = 8.8, 3.4 Hz, 1H), 6.76 (s, 1H), (d, *J* = 4.0 Hz), 137.3, 134.6 (d, *J* = 15.7 Hz), 128.8 (d, *J* = 7.3 Hz), 125.8, 109.7 (d, *J* = 40.3 Hz). ^19^F NMR (376 MHz, Acetone-*d*_6_) δ −58.8. ^13^C NMR (126 MHz, acetone-*d*_6_) δ 169.3, 158.3 (d, *J* = 227.2 Hz), 153.1, 145.7, 141.9 HRMS: (ESI) [M – H]^−^ calcd for C_10_H_6_FN_5_O_2_, 248.0579, observed 248.0578 *m*/*z*. Δ −0.4031 ppm.

#### 6-(6-(Trifluoromethyl)pyridin-3-yl)-[1,2,5]oxadiazolo[3,4-*b*]pyridin-7-ol (**7l**)

Yellow solid (36%, 67 mg) ^1^H NMR (500 MHz, acetone-*d*_6_) δ 11.94 (s, 1H), 8.98 (d, *J* = 2.2 Hz, 1H), 8.55 (s, 1H), 8.35 (ddd, *J* = 8.2, 2.2, 0.8 Hz, 1H), 7.91 (dd, *J* = 8.2, 0.8 Hz, 1H). ^19^F NMR (376 MHz, acetone) δ −68.30. ^13^C NMR (126 MHz, acetone-*d*_6_) δ 170.9, 153.7, 150.4, 146.8 (d, *J* = 34.5 Hz), 146.0, 144.3, 138.5, 134.4, 122.9 (d, *J* = 273.2 Hz), 120.8 (d, *J* = 2.9 Hz), 118.9. HRMS: (ESI) [M – H]^−^ calcd for C_11_H_5_F_3_N_4_O_2_, 283.0437, observed 283.0437 *m*/*z*. Δ 0.0 ppm.

#### 6-((2-Fluorophenyl)amino)-[1,2,5]oxadiazolo[3,4-*b*]pyridin-7-ol (**7m**)

Purple solid (21%, 35 mg) ^1^H NMR (400 MHz, acetone-*d*_6_) δ 11.34 (s, 1H), 8.18 (d, *J* = 1.3 Hz, 1H), 7.14–6.99 (m, 2H), 6.85–6.75 (m, 1H), 6.28 (s, 1H). ^19^F NMR (376 MHz, acetone-*d*_6_) δ −134.3. ^13^C NMR (126 MHz, acetone-*d*_6_) δ 153.7 (d, *J* = 148.2 Hz), 152.4, 145.5, 136.8, 134.9, 125.5, 125.4 (d, *J* = 3.5 Hz), 120.4 (d, *J* = 6.9 Hz), 116.9 (d, *J* = 2.8 Hz), 115.8 (d, *J* = 19.6 Hz). HRMS: (ESI) [M – H]^−^ calcd for C_11_H_7_FN_4_O_2_, 245.0480, observed 245.0480 *m*/*z*. Δ 0.0 ppm.

#### 6-((3-Fluorophenyl)amino)-[1,2,5]oxadiazolo[3,4-*b*]pyridin-7-ol (**7n**)

Red solid (23%, 36 mg) ^1^H NMR (400 MHz, acetone-*d*_6_) δ 11.34 (s, 1H), 8.23 (d, *J* = 0.6 Hz, 1H), 7.15 (td, *J* = 8.2, 6.8 Hz, 1H), 6.77 (ddd, *J* = 8.2, 2.2, 0.9 Hz, 2H), 6.68 (dt, *J* = 11.9, 2.3 Hz, 1H), 6.46 (dddd, *J* = 8.9, 8.1, 2.5, 0.9 Hz, 1H). ^19^F NMR (376 MHz, acetone-*d*_6_) δ −114.6. ^13^C NMR (126 MHz, acetone-*d*_6_) δ 169.49, 164.8 (d, *J* = 240.9 Hz), 153.2, 149.7 (d, *J* = 8.9 Hz), 145.8, 138.3, 131.2 (d, *J* = 10.0 Hz), 125.3, 111.6, 105.7 (d, *J* = 21.5 Hz), 102.0 (d, *J* = 25.4 Hz). HRMS: (ESI) [M – H]^−^ calcd for C_11_H_7_FN_4_O_2_, 245.0480 observed 245.0471 *m*/*z*. Δ −3.6727 ppm.

#### 6-((4-Fluorophenyl)amino)-[1,2,5]oxadiazolo[3,4-*b*]pyridin-7-ol (**7o**)

Purple solid (11%, 11 mg) ^1^H NMR (500 MHz, acetone-*d*_6_) δ 11.26 (s,1 H), 8.14 (s, 1H), 7.09–6.89 (m, 4H), 6.56 (s, 1H). ^19^F NMR (376 MHz, acetone-*d*_6_) δ −126.6 (tt, *J* = 8.8, 4.7 Hz). ^13^C NMR (126 MHz, acetone-*d*_6_) δ 168.7, 157.67 (d, *J* = 235.4 Hz), 152.9, 145.3, 142.9, 134.6, 127.2, 118.1 (d, *J* = 7.6 Hz), 116.2 (d, *J* = 22.5 Hz). HRMS: (ESI) [M – H]^−^ calcd for C_11_H_7_FN_4_O_2_, 247.0625, observed 247.0626 *m*/*z*. Δ 0.4047 ppm.

#### 6-((2-Chlorophenyl)amino)-[1,2,5]oxadiazolo[3,4-*b*]pyridin-7-ol (**7p**)

Yellow solid (62%, 56 mg) ^1^H NMR (400 MHz, acetone-*d*_6_) δ 11.37 (s, 1H), 8.26 (d, *J* = 0.7 Hz, 1H), 7.34 (dd, *J* = 7.9, 1.6 Hz, 1H), 7.11 (dddd, *J* = 7.9, 7.3, 1.5, 0.5 Hz, 1H), 6.96 (dd, *J* = 8.2, 1.6 Hz, 1H), 6.78 (ddd, *J* = 7.9, 7.3, 1.5 Hz, 1H). ^13^C NMR (126 MHz, acetone-*d*_6_) δ 153.4, 145.8, 143.3, 139.1, 130.3, 128.7, 124.8, 121.0, 120.5, 115.4. HRMS: (ESI) [M + H]^+^ calcd for C_11_H_7_ClN_4_O_2_ 263.0330 observed 263.0340 *m*/*z*. Δ 3.6877 ppm.

#### 6-((2-(Trifluoromethyl)phenyl)amino)-[1,2,5]oxadiazolo[3,4-*b*]pyridin-7-ol (**7q**)

Purple solid (35%, 44 mg) ^1^H NMR (400 MHz, acetone-*d*_6_) δ 11.47 (s, 1H), 8.26 (s, 1H), 7.56 (dd, *J* = 8.0, 1.6 Hz, 1H), 7.38 (t, *J* = 7.4 Hz, 1H), 7.10 (s, 1H), 6.92 (ddt, *J* = 8.1, 7.2, 1.0 Hz, 1H), 6.42 (s, 1H). ^19^F NMR (376 MHz, acetone-*d*_6_) δ −62.4. ^13^C NMR (126 MHz, acetone-*d*_6_) δ 169.1, 153.3, 145.7, 145.1, 139.3, 134.0, 127.3 (q, *J* = 5.4 Hz), 126.1 (d, *J* = 271.6 Hz), 124.5, 119.5, 116.6, 116.0 (d, *J* = 29.4 Hz). HRMS: (ESI) [M – H]^−^ calcd for C_12_H_7_F_3_N_4_O_2_, 295.0448, observed 295.0442 *m*/*z*. Δ −2.0335 ppm.

#### 6-((3-(Trifluoromethyl)phenyl)amino)-[1,2,5]oxadiazolo[3,4-*b*]pyridin-7-ol (**7r**)

Red solid (56%) ^1^H NMR (400 MHz, acetone-*d*_6_) δ 11.37 (s, 1H), 8.28 (d, *J* = 0.7 Hz, 1H), 7.34 (t, *J* = 8.1, 7.6 Hz, 1H), 7.23–7.10 (m, 2H), 7.08–6.99 (m, 1H), 6.9 3(s, 1H). ^19^F NMR (376 MHz, acetone-*d*_6_) δ −63.3. ^13^C NMR (126 MHz, acetone-*d*_6_) δ 153.3, 148.5, 145.9, 139.4, 131.7 (d, *J* = 31.5 Hz), 130.6, 125.5 (d, *J* = 271.9 Hz), 124.8 (d, *J* = 265.2 Hz), 118.7, 115.5 (q, *J* = 4.3 Hz), 111.5 (q, *J* = 4.3 Hz). HRMS: (ESI) [M – H]^−^ calcd for C_12_H_7_F_3_N_4_O_2_, 295.0448, observed 295.0440 *m*/*z*. Δ −2.7114 ppm.

#### 6-((4-(Trifluoromethyl)phenyl)amino)-[1,2,5]oxadiazolo[3,4-*b*]pyridin-7-ol (**7s**)

Red solid (84%, 78 mg) ^1^H NMR (500 MHz, acetone-*d*_6_) δ 11.45 (s, 1H), 8.29 (s, 1H), 7.43 (d, *J* = 8.4 Hz, 2H), 7.09 (s, 1H), 7.01 (d, *J* = 8.4 Hz, 2H). ^19^F NMR (376 MHz, acetone-*d*_6_) δ −61.5. ^13^C NMR (126 MHz, acetone-*d*_6_) δ 169.56, 153.34, 151.18, 146.00, 140.18, 127.09 (d, *J* = 4.1 Hz), 125.74 (d, *J* = 271.4 Hz), 124.29, 120.05 (d, *J* = 32.4 Hz), 114.63. HRMS: (ESI) [M – H]^−^ calcd for C_12_H_7_F_3_N_4_O_2_, 295.0443, observed 295.0450 *m*/*z*. Δ 2.4 ppm.

#### 6-((2,3-Difluorophenyl)amino)-[1,2,5]oxadiazolo[3,4-*b*]pyridin-7-ol (**7t**)

Purple solid (38%, 66 mg) ^1^H NMR (400 MHz, acetone-*d*_6_) δ 11.43 (s, 1H), 8.24 (d, *J* = 0.8 Hz, 1H), 6.92 (dddd, *J* = 11.0, 8.6, 5.7, 2.3 Hz, 1H), 6.78–6.56 (m, 3H). ^19^F NMR (376 MHz, acetone-*d*_6_) δ −141.8, −161.1. ^13^C NMR (126 MHz, acetone-*d*_6_) δ 169.8, 153.8, 152.3 (dd, *J* = 242.8, 10.5 Hz), 146.4, 141.6 (dd, *J* = 240.2, 15.3 Hz), 140.2, 138.2 (d, *J* = 8.1 Hz), 125.3 (dd, *J* = 8.8, 4.8 Hz), 124.9, 112.2, 107.6 (d, *J* = 17.5 Hz). HRMS: (ESI) [M – H]^−^ calcd for C_11_H_6_F_2_N_4_O_2_ 263.0386 observed 263.0387 *m*/*z*. Δ 0.3801 ppm.

#### 6-((2,4-Difluorophenyl)amino)-[1,2,5]oxadiazolo[3,4-*b*]pyridin-7-ol (**7u**)

Purple solid (17%, 29 mg) ^1^H NMR (500 MHz, acetone-*d*_6_) δ 11.31 (s, 1H), 8.11 (s, 1H), 7.1–7.04 (m, 2H), 6.87–6.76 (m, 1H), 6.36–6.28 (m, 1H). ^19^F NMR (376 MHz, acetone-*d*_6_) δ −123.7 (dd, *J* = 8.8, 6.1 Hz), −128.6 (t, *J* = 10.9 Hz). ^13^C NMR (126 MHz, acetone) δ 157.1 (dd, *J* = 238.8, 11.0 Hz), 153.3 (dd, *J* = 243.7, 12.0 Hz), 153.2, 145.54, 136.2, 132.5–130.0 (m), 126.2, 118.6 (dd, *J* = 9.0, 4.0 Hz), 111.7 (dd, *J* = 21.8, 3.7 Hz), 104.63 (dd, *J* = 26.9, 23.3 Hz). HRMS: (ESI) [M – H]^−^ calcd for C_11_H_6_F_2_N_4_O_2_ 263.0386 observed 263.0388 *m*/*z*. Δ 0.7603 ppm.

#### 6-((2,5-Difluorophenyl)amino)-[1,2,5]oxadiazolo[3,4-*b*]pyridin-7-ol (**7v**)

Purple solid (43%, 74 mg) ^1^H NMR (400 MHz, acetone-*d*_6_) δ 11.44 (s, 1H), 8.27 (d, *J* = 0.8 Hz, 1H), 7.06 (ddd, *J* = 11.3, 8.9, 5.1 Hz, 1H), 6.66 (ddd, *J* = 10.4, 7.1, 3.1 Hz, 1H), 6.43 (ddt, *J* = 8.9, 8.1, 3.3 Hz, 1H). ^19^F NMR (376 MHz, acetone-*d*_6_) δ −119.4 to −119.6 (m), −141.2. ^13^C NMR (126 MHz, acetone-*d*_6_) δ 169.5, 160.4 (d, *J* = 243.6 Hz), 153.4, 148.6 (d, *J* = 243.6 Hz), 146.1, 140.6, 138.2–135.8 (m), 123.8, 116.2 (dd, *J* = 21.3, 10.5 Hz), 104.5 (dd, *J* = 24.8, 7.4 Hz), 102.7 (dd, *J* = 29.0, 3.4 Hz). HRMS: (ESI) [M – H]^−^ calcd for C_11_H_6_F_2_N_4_O_2_, 263.03861, observed 263.0377 *m*/*z*. Δ −3.4595 ppm.

#### 6-((2,6-Difluorophenyl)amino)-[1,2,5]oxadiazolo[3,4-*b*]pyridin-7-ol (**7w**)

Red solid (22% yield, 23 mg) ^1^H NMR (500 MHz, acetone-*d*_6_) δ 11.20 (s, 1H), 7.69 (s, 1H), 7.14–6.99 (m, 3H), 6.37 (s, 1H). ^13^C NMR (126 MHz, acetone-*d*_6_) δ 167.37, 156.77 (dd, *J* = 244.8, 6.2 Hz), 152.56, 144.51, 129.07, 128.54, 123.59 (t, *J* = 9.7 Hz), 121.34 (t, *J* = 15.0 Hz), 112.77 (dd, *J* = 18.1, 5.5 Hz). HRMS: (ESI) [M – H]^+^ calcd for C_11_H_6_F_2_N_4_O_2_ 265.0532 observed 265.0533 *m*/*z*. Δ 0.3772 ppm.

#### 6-((3,4-Difluorophenyl)amino)-[1,2,5]oxadiazolo[3,4-*b*]pyridin-7-ol (**7x**)

Purple solid (57%, 98 mg) ^1^H NMR (400 MHz, acetone-*d*_6_) δ 11.36 (s, 1H), 8.22 (d, *J* = 0.6 Hz, 1H), 7.09 (dt, *J* = 10.6, 9.0 Hz, 1H), 6.86 (ddd, *J* = 13.2, 6.9, 2.8 Hz, 1H), 6.76–6.69 (m, 2H). ^19^F NMR (376 MHz, acetone-*d*_6_) δ −139.6, −153.4. ^13^C NMR (126 MHz, acetone-*d*_6_) δ 169.4, 153.2, 151.3 (dd, *J* = 242.8, 13.5 Hz), 145.8, 144.9 (d, *J* = 8.4 Hz), 144.3 (dd, *J* = 235.9, 12.9 Hz), 138.2, 125.6, 118.1 (d, *J* = 18.0 Hz), 111.5 (d, *J* = 2.6 Hz), 104.2 (d, *J* = 20.9 Hz). HRMS: (ESI) [M – H]^−^ calcd for C_11_H_6_F_2_N_4_O_2_ 263.0386 observed 263.0387 *m*/*z*. Δ 0.3801 ppm.

#### 6-((2,3,4-Trifluorophenyl)amino)-[1,2,5]oxadiazolo[3,4-*b*]pyridin-7-ol (**7y**)

Purple solid (22%, 26 mg) ^1^H NMR (400 MHz, acetone-*d*_6_) δ 8.20 (s, 1H), 6.94 (dddd, *J* = 10.3, 9.4, 8.1, 2.4 Hz, 1H), 6.73 (tdd, *J* = 9.3, 4.9, 2.5 Hz, 1H), 6.56 (s, 1H). ^19^F NMR (376 MHz, acetone-*d*_6_) δ −148.7 to −152.1 (m), −153.3 to −157.4 (m), −163.7 (td, *J* = 20.7, 19.9, 7.7 Hz). ^13^C NMR (126 MHz, acetone-*d*_6_) δ 169.2, 153.3, 145.8, 145.0 (dd, *J* = 238.7, 9.3 Hz), 142.1 (dd, *J* = 244.4, 12.8 Hz), 140.9 (dd, *J* = 245.5, 5.2 Hz), 139.2, 133.5 (d, *J* = 8.7 Hz), 124.6, 112.1 (dd, *J* = 17.7, 3.8 Hz), 110.6 (dt, *J* = 6.8, 3.3 Hz). HRMS: (ESI) [M + H]^+^ calcd for C_11_H_5_F_3_N_4_O_2_ 283.0437 observed 283.0441 *m*/*z*. Δ 1.4132 ppm.

#### 6-((Perfluorophenyl)amino)-[1,2,5]oxadiazolo[3,4-*b*]pyridin-7-ol (**7z**)

Orange solid (58%, 91 mg) ^1^H NMR (500 MHz, DMSO) δ 12.75 (s, 1H), 8.07 (s, 1H), 7.68 (s, 1H). ^19^F NMR (376 MHz, acetone) δ −155.6 (d, *J* = 22.3 Hz), −166.7 (td, *J* = 20.8, 4.1 Hz), −170.4 (tt, *J* = 21.4, 4.4 Hz). ^13^C NMR (126 MHz, DMSO) δ 167.1, 151.9, 144.3, 138.8 (d, *J* = 241.2 Hz), 138.4, 136.4, 133.6 (d, *J* = 241.4 Hz), 124.5, 121.3. HRMS: (ESI) [M – H]^−^ calcd for C_11_H_3_F_5_N_4_O_2_, 317.01034, observed 317.0095 *m*/*z*. Δ −2.6497 ppm.

#### 6-((3-Chloro-2-fluorophenyl)amino)-[1,2,5]oxadiazolo[3,4-*b*]pyridin-7-ol (**7aa**)

Red solid (58%, 116 mg) ^1^H NMR (400 MHz, Acetone-*d*_6_) δ 11.43 (s, 1H), 8.25 (d, *J* = 0.8 Hz, 1H), 6.97–6.78 (m, 3H), 6.62 (s, 1H). ^19^F NMR (376 MHz, acetone-*d*_6_) δ −137.6. ^13^C NMR (126 MHz, acetone-*d*_6_) δ 169.3, 153.3, 148.4 (d, *J* = 241.4 Hz), 145.9, 140.1, 137.2 (d, *J* = 10.6 Hz), 125.6 (d, *J* = 4.7 Hz), 124.2, 121.0 (d, *J* = 15.1 Hz), 120.0, 114.9. HRMS: (ESI) [M + H]^+^ calcd for 281.0236 observed 281.0241 *m*/*z*. Δ 1.7436 ppm.

#### 6-((2-Fluoro-3-(trifluoromethyl)phenyl)amino)-[1,2,5]oxadiazolo[3,4-*b*]pyridin-7-ol (**7ab**)

Red solid (55%, 112 mg) ^1^H NMR (500 MHz, acetone-*d*_6_) δ 11.50 (s, 1H), 8.30 (d, *J* = 0.8 Hz, 1H), 7.24–7.08 (m, 2H), 7.02 (td, *J* = 7.3, 1.3 Hz, 1H), 6.80 (s, 1H). ^19^F NMR (376 MHz, acetone-*d*_6_) δ −61.6 (d, *J* = 13.0 Hz), −137.1 to −140.8 (m). ^13^C NMR (126 MHz, acetone-*d*_6_) δ 169.5, 153.5, 149.4 (d, *J* = 248.0 Hz), 146.1, 141.2, 137.2 (d, *J* = 9.9 Hz), 125.3 (d, *J* = 4.4 Hz), 124.1 (q, *J* = 271.4 Hz), 123.6, 120.1 (d, *J* = 4.0 Hz), 118.3 (qd, *J* = 33.2, 32.6, 10.2 Hz), 115.7 (q, *J* = 4.7 Hz). HRMS: (ESI) [M – H]^−^ calcd for C_12_H_6_F_4_N_4_O_2_ 313.03541 observed 313.0348 *m*/*z*. Δ −1.9486 ppm.

#### 6-((2-Fluoro-5-(trifluoromethyl)phenyl)amino)-[1,2,5]oxadiazolo[3,4-*b*]pyridin-7-ol (**7ac**)

Purple solid (43%, 67 mg) ^1^H NMR (400 MHz, acetone-*d*_6_) δ 11.48 (s, 1H), 8.34 (d, *J* = 0.7 Hz, 1H), 7.26 (dddd, 1H), 7.15 (dd, *J* = 8.1, 1.4 Hz, 1H), 7.12–7.0 (m, 1H), 6.78–6.60 (m, 1H). ^19^F NMR (376 MHz, acetone-*d*_6_) δ −62.5, −130.1 (d, *J* = 12.0 Hz) ^13^C NMR (126 MHz, acetone-*d*_6_) δ 169.7, 154.3 (d, *J* = 245.3 Hz), 153.4, 146.1, 141.6, 136.9 (d, *J* = 11.9 Hz), 127.3 (qd, *J* = 32.2, 3.4 Hz), 125.1 (q, *J* = 271.3 Hz), 123.3, 116.3, 116.1, 112.5 (t, *J* = 4.0 Hz).HRMS: (ESI) [M – H]^−^ calcd for C_12_H_6_F_4_N_4_O_2_, 313.0354, observed 313.0350 *m*/*z*. Δ −1.2778 ppm.

#### 6-((2-Fluoro-6-(trifluoromethyl)phenyl)amino)-[1,2,5]oxadiazolo[3,4-*b*]pyridin-7-ol (**7ad**)

Orange solid (32%, 34 mg) ^1^H NMR (500 MHz, DMSO) δ 12.51 (s, 1H), 7.70 (s, 1H), 7.53–7.43 (m, 2H), 7.28–7.14 (m, 1H), 6.86 (s, 1H). ^19^F NMR (376 MHz, DMSO) δ −58.9, −116.3 (d, *J* = 11.3 Hz). ^13^C NMR (126 MHz, DMSO) δ 165.8, 156.2 (d, *J* = 246.6 Hz), 151.5, 143.9, 131.1, 130.2 (d, *J* = 12.6 Hz), 127.8, 123.9 (q, *J* = 272.8 Hz), 123.5, 122.6, 120.7 (d, *J* = 20.4 Hz). HRMS: (ESI) [M – H]^−^ calcd for C_12_H_6_F_4_N_4_O_2_, 313.0354, observed 313.0345 *m*/*z*. Δ −2.8750 ppm.

#### 6-((2,4-Difluoro-5-(trifluoromethyl)phenyl)amino)-[1,2,5]oxadiazolo[3,4-*b*]pyridin-7-ol (**7ae**)

Yellow solid (31%, 67 mg) ^1^H NMR (500 MHz, acetone-*d*_6_) δ 11.54 (s, 1H), 8.29 (s, 1H), 7.29 (t, *J* = 10.8 Hz, 1H), 7.15 (dd, *J* = 9.1, 7.1 Hz, 1H), 6.72 (s, 1H). ^19^F NMR (376 MHz, acetone-*d*_6_) δ −61.1 (d, *J* = 13.3 Hz), −123.9, −126.8. ^13^C NMR (126 MHz, acetone-*d*_6_) δ 169.6, 154.6 (dd, *J* = 182.9, 10.9 Hz), 153.4, 152.8 (dd, *J* = 180.7, 14.3 Hz), 146.1, 140.7, 133.2 (d, *J* = 11.4 Hz), 123.8, 123.7 (q, *J* = 273.7, 271.6, 270.5 Hz), 113.6 (d, *J* = 5.0 Hz), 106.2 (t, *J* = 24.7 Hz). HRMS: (ESI) [M – H]^−^ calcd for C_12_H_5_F_5_N_4_O_2_ 331.0259 observed 331.0252 *m*/*z*. Δ −2.1146 ppm.

#### 6-((5-Fluoro-2-(trifluoromethyl)phenyl)amino)-[1,2,5]oxadiazolo[3,4-*b*]pyridin-7-ol (**7af**)

Purple solid (40%) ^1^H NMR (400 MHz, acetone-*d*_6_) δ 11.52 (s, 1H), 8.33 (d, *J* = 0.7 Hz, 1H), 7.58 (dd, *J* = 6.2, 0.7 Hz, 1H), 6.77 (dd, *J* = 11.9, 0.8 Hz, 1H), 6.65–6.56 (m, 2H). ^13^C NMR (126 MHz, acetone-*d*_6_) δ 169.7, 154.3 (d, *J* = 245.3 Hz), 153.4, 146.1, 141.6, 136.9 (d, *J* = 11.9 Hz), 127.3 (qd, *J* = 32.2, 3.4 Hz), 125.1 (q, *J* = 271.3 Hz), 123.3, 116.3, 116.1, 112.5 (t, *J* = 4.0 Hz). HRMS: (ESI) [M – H]^−^ calcd for C_12_H_6_F_4_N_4_O_2_, 313.0354, observed 313.0350 *m*/*z*. Δ −1.2778 ppm.

#### 6-((3-Fluoro-5-(trifluoromethyl)phenyl)amino)-[1,2,5]oxadiazolo[3,4-*b*]pyridin-7-ol (**7ag**)

Orange solid (24% yield, 78 mg) ^1^H NMR (500 MHz, Acetone-*d*_6_) δ 11.48 (s, 1H), 8.32 (d, *J* = 0.7 Hz, 1H), 7.22 (s, 1H), 7.03 (s, 1H), 6.86 (dt, *J* = 11.5, 2.2 Hz, 1H), 6.73 (d, *J* = 8.7 Hz, 1H). ^19^F NMR (376 MHz, acetone-*d*_6_) δ −63.6, −112.3 (t, *J* = 10.3 Hz). ^13^C NMR (126 MHz, acetone-*d*_6_) δ 169.9, 164.6 (d, *J* = 242.9 Hz), 153.6 (d, *J* = 7.8 Hz), 151.5, 151.4, 146.3, 141.6, 133.1 (dd, *J* = 32.6, 10.3 Hz), 123.7, 108.8–106.5 (m), 104.6 (d, *J* = 25.8 Hz), 102.0 (dd, *J* = 25.7, 4.0 Hz). HRMS: (ESI) [M – H]^−^ calcd for C_12_H_6_F_4_N_4_O_2_, 313.0354, observed 313.035 *m*/*z*. Δ −1.2778

#### 6-((3-Fluoro-[1,1′-biphenyl]-4-yl)amino)-[1,2,5]oxadiazolo[3,4-*b*]pyridin-7-ol (**7ah**)

Purple solid (48%, 71 mg) ^1^H NMR (400 MHz, acetone-*d*_6_) δ 8.25 (s, 1H), 7.63–7.59 (m, 2H), 7.45–7.43 (m, 1H), 7.42–7.39 (m, 2H), 7.33–7.27 (m, 2H), 7.08 (t, 1H, *J* = 9.0), 6.51 (s, 1H). ^19^F NMR (376 MHz, acetone-*d*_6_) δ −133.95 to −134.09 (m). ^13^C NMR (126 MHz, acetone-*d*_6_) δ 152.6 (d, *J* = 239.8 Hz), 152.3, 144.7, 139.7, 139.7, 136.5, 133.4 (d, *J* = 11.8 Hz), 132.4 (d, *J* = 7.0 Hz), 128.8, 126.8, 126.2, 124.3, 122.7, 122.7, 116.1, 116.1, 113.3 (d, *J* = 19.9 Hz). HRMS: (ESI) [M – H]^−^ calcd for C_17_H_11_FN_4_O_2_, 321.0793, observed 321.0790 *m*/*z*. Δ −1.0278 ppm.

#### 6-([1,1′-Biphenyl]-3-ylamino)-[1,2,5]oxadiazolo[3,4-*b*]pyridin-7-ol (**7ai**)

Yellow solid (46%, 110 mg) ^1^H NMR (500 MHz, acetone-*d*_6_) δ 11.28 (s, 1H), 8.27 (s, 1H), 7.63–7.55 (m, 2H), 7.41 (t, *J* = 7.6 Hz, 2H), 7.32 (tt, *J* = 7.7, 1.3 Hz, 1H), 7.28 (t, *J* = 1.7 Hz, 1H), 7.26 (d, *J* = 7.8 Hz, 1H), 7.07 (ddd, *J* = 7.6, 1.7, 1.0 Hz, 1H), 7.01 (ddd, *J* = 8.1, 2.3, 0.9 Hz, 1H), 6.68 (s, 1H). ^13^C NMR (126 MHz, acetone-*d*_6_) δ 168.9, 152.9, 147.1, 145.4, 142.9, 142.2, 130.4, 129.5, 128.1, 127.7, 126.6, 118.9, 115.4, 114.8. HRMS: (ESI) [M – H]^−^ calcd for C_17_H_12_N_4_O_2_ 303.0887 observed 303.0881 *m*/*z*. Δ −1.9796 ppm.

#### 6-([1,1′-Biphenyl]-2-ylamino)-[1,2,5]oxadiazolo[3,4-*b*]pyridin-7-ol (**7aj**)

Yellow solid (16%, 34 mg) ^1^H NMR (500 MHz, acetone-*d*_6_) δ 12.00 (s, 1H), 8.87 (s, 1H), 8.32 (dd, *J* = 8.2, 1.4 Hz, 2H), 8.25 (t, *J* = 7.7 Hz, 2H), 8.19–8.09 (m, 1H), 8.04–7.95 (m, 2H), 7.91 (dd, *J* = 8.2, 1.2 Hz, 1H), 7.74 (td, *J* = 7.4, 1.2 Hz, 1H), 6.82 (s, 1H). ^13^C NMR (126 MHz, acetone-*d*_6_) δ 168.6, 152.8, 145.0, 142.7, 140.2, 134.0, 131.5, 130.0, 129.7, 129.2, 128.2, 127.0, 121.3, 116.6. HRMS: (ESI) [M – H]^−^ calcd for C_17_H_12_N_4_O_2_ 303.0887 observed 303.0885 *m*/*z*. Δ −0.6599 ppm.

#### 6-((2′-Fluoro-[1,1′-biphenyl]-4-yl)amino)-[1,2,5]oxadiazolo[3,4-*b*]pyridin-7-ol (**7ak**)

Orange solid (40%, 83 mg) ^1^H NMR (500 MHz, acetone-*d*_6_) δ j11.31 (s, 1H), 8.27 (s, 1H), 7.48 (td, *J* = 7.9, 1.9 Hz, 1H), 7.41 (dd, *J* = 8.7, 1.8 Hz, 2H), 7.32 (dddd, *J* = 8.1, 7.0, 5.0, 1.8 Hz, 1H), 7.24 (dd, *J* = 7.5, 1.3 Hz, 1H), 7.23–7.15 (m, 1H), 7.12–7.02 (m, 2H), 6.75 (s, 1H). ^19^F NMR (376 MHz, acetone-*d*_6_) δ −119.7. ^13^C NMR (126 MHz, acetone-*d*_6_) δ 160.6 (d, *J* = 245.1 Hz), 153.1, 146.6, 145.5, 136.3, 131.2 (d, *J* = 3.7 Hz), 130.5 (d, *J* = 3.2 Hz), 129.8 (d, *J* = 13.1 Hz), 129.1 (d, *J* = 8.3 Hz), 127.1, 126.1, 125.5 (d, *J* = 3.6 Hz), 116.7 (d, *J* = 23.0 Hz), 115.9. HRMS: (ESI) [M – H]^+^ calcd for C_17_H_11_FN_4_O_2_, 323.09388, observed 323.0942 *m*/*z*. Δ 0.9904 ppm.

#### 6-((3′-Fluoro-[1,1′-biphenyl]-4-yl)amino)-[1,2,5]oxadiazolo[3,4-*b*]pyridin-7-ol (**7al**)

Purple solid (25%, 53 mg) ^1^H NMR (500 MHz, acetone-*d*_6_) δ 11.33 (s, 1H), 8.26 (s, 1H), 7.53 (d, *J* = 8.7 Hz, 2H), 7.46–7.40 (m, 2H), 7.34 (ddt, *J* = 10.5, 2.1, 1.1 Hz, 1H), 7.13–7.05 (m, 2H), 7.06–6.97 (m, 1H), 6.77 (s, 1H). ^19^F NMR (376 MHz, acetone-*d*_6_) δ −114.9. ^13^C NMR (126 MHz, acetone-*d*_6_) δ 164.2 (d, *J* = 243.0 Hz), 153.1, 147.0, 145.5, 144.4 (d, *J* = 8.0 Hz), 136.6, 131.3 (d, *J* = 8.6 Hz), 131.0 (d, *J* = 2.3 Hz), 128.5, 126.0, 122.7 (d, *J* = 2.5 Hz), 116.3, 113.6 (d, *J* = 21.4 Hz), 113.3 (d, *J* = 22.2 Hz). HRMS: (ESI) [M – H]^−^ calcd for C_17_H_11_FN_4_O_2_, 323.0938, observed 323.0953 *m*/*z*. Δ 4.6426 ppm.

#### 6-((3′-Chloro-[1,1′-biphenyl]-4-yl)amino)-[1,2,5]oxadiazolo[3,4-*b*]pyridin-7-ol (**7am**)

Purple Solid (9%, 19 mg) ^1^H NMR (400 MHz, acetone-*d*_6_) δ 11.30 (s, 1H), 8.28–8.23 (m, 1H), 7.61–7.58 (m, 1H), 7.57–7.49 (m, 3H), 7.41 (td, *J* = 7.9, 0.4 Hz, 1H), 7.28 (ddd, *J* = 7.9, 2.1, 1.0 Hz, 1H), 7.09 (d, *J* = 8.7 Hz, 1H), 6.77 (s, 1H). ^13^C NMR (126 MHz, acetone-*d*_6_) δ 153.0, 147.1, 145.5, 144.0, 136.5, 135.1, 131.2, 130.8, 128.5, 126.9, 126.7, 125.9 (d, *J* = 8.6 Hz), 125.4, 116.3, 116.3. HRMS: (ESI) [M – H]^−^ calcd for C_17_H_11_ClN_4_O_2_, 337.0497, observed 337.0492 *m*/*z*. Δ −1.4834 ppm.

#### 6-((3′-(Trifluoromethyl)-[1,1′-biphenyl]-4-yl)amino)-[1,2,5]oxadiazolo[3,4-*b*]pyridin-7-ol (**7an**)

Purple solid (33%, 82 mg) ^1^H NMR (500 MHz, acetone-*d*_6_) δ 11.34 (s, 1H), 8.27 (s, 1H), 7.95–7.83 (m, 2H), 7.67–7.43 (m, 4H), 7.10 (d, *J* = 8.7 Hz, 2H), 6.80 (s, 1H). ^19^F NMR (376 MHz, acetone-*d*_6_) δ −63.1. ^13^C NMR (126 MHz, acetone-*d*_6_) δ 153.1, 147.3, 145.6, 142.9, 136.8, 131.4 (d, *J* = 31.6 Hz), 130.6 (d, *J* = 3.6 Hz), 130.5, 128.6, 125.9, 125.5 (d, *J* = 271.5 Hz), 123.4 (dd, *J* = 37.7, 4.0 Hz), 116.3. HRMS: (ESI) [M + H]^+^ calcd for C_18_H_11_F_3_N_4_O_2_ 373.0906 observed 373.0918 *m*/*z*. Δ 3.2163 ppm.

#### 6-((4′-Fluoro-[1,1′-biphenyl]-4-yl)amino)-[1,2,5]oxadiazolo[3,4-*b*]pyridin-7-ol (**7ao**)

Purple solid (17%, 34 mg) ^1^H NMR (400 MHz, acetone-*d*_6_) δ 11.31 (s, 1H), 8.24 (s, 1H), 7.61 (dd, *J* = 8.9, 5.4 Hz, 2H), 7.48 (d, *J* = 8.7 Hz, 2H), 7.16 (t, *J* = 8.9 Hz, 2H), 7.09 (d, *J* = 8.7 Hz, H), 6.70 (s, 1H). ^19^F NMR (376 MHz, acetone-*d*_6_) δ −118.9. ^13^C NMR (126 MHz, acetone-*d*_6_) δ 162.7 (d, *J* = 243.3 Hz), 152.9, 146.3, 145.4, 138.3, 135.5, 131.7, 128.7 (d, *J* = 7.9 Hz), 128.3, 126.4, 116.5, 116.2 (d, *J* = 21.4 Hz). HRMS: (ESI) [M – H]^−^ calcd for C_17_H_11_FN_4_O_2_ 321.0793 observed 321.0791 *m*/*z*. Δ −0.6228 ppm.

#### 6-((4′-Chloro-[1,1′-biphenyl]-4-yl)amino)-[1,2,5]oxadiazolo[3,4-*b*]pyridin-7-ol (**7ap**)

Purple solid (42%, 1.98 g) ^1^H NMR (400 MHz, acetone-*d*_6_) δ 11.31 (s, 1H), 8.23 (s, 1H), 7.60 (d, *J* = 8.5 Hz, 2H), 7.49 (d, *J* = 8.5 Hz, 3H), 7.41 (d, *J* = 8.5 Hz, 3H), 7.07 (d, *J* = 8.7 Hz, 3H), 6.72 (s, 1H). ^13^C NMR (126 MHz, acetone-*d*_6_) δ 169.26, 153.00, 146.85, 145.58, 140.68, 136.12, 132.56, 131.12, 129.66, 128.53, 128.41, 126.19, 116.40. HRMS: ESI [M – H]^−^ calc for C_17_H_11_ClN_4_O_2_, 339.0643, observed, 339.0646. *m*/*z*. Δ 0.8847 ppm.

#### 6-((4′-(Trifluoromethyl)-[1,1′-biphenyl]-4-yl)amino)-[1,2,5]oxadiazolo[3,4-*b*]pyridin-7-ol (**7aq**)

Purple solid (30%, 73 mg) ^1^H NMR (400 MHz, acetone-*d*_6_) δ 11.33 (s, 1H), 8.27 (d, *J* = 0.6 Hz, 1H), 7.86–7.78 (m, 2H), 7.73 (d, *J* = 8.6 Hz, 2H), 7.58 (d, *J* = 8.7 Hz, 2H), 7.10 (d, *J* = 8.8 Hz, 1H), 6.82 (s, 1H). ^19^F NMR (376 MHz, acetone-*d*_6_) δ −62.8. ^13^C NMR (126 MHz, acetone-*d*_6_) δ 169.1, 153.1, 147.6, 145.6 (d, *J* = 16.8 Hz), 137.0, 130.5, 128.7, 128.4 (d, *J* = 31.9 Hz), 127.3, 126.5 (d, *J* = 4.0 Hz), 125.8, 125.7, 125.6 (d, *J* = 271.1 Hz), 116.2. HRMS: (ESI) [M + H]^+^ calcd for C_18_H_11_F_3_N_4_O_2_ 373.0907 observed 373.0906 *m*/*z*. Δ −0.2680 ppm.

#### 6-((4-(6-(Trifluoromethyl)pyridin-3-yl)phenyl)amino)-[1,2,5]oxadiazolo[3,4-*b*]pyridin-7-ol (**7ar**)

Purple solid (42%, 71 mg) ^1^H NMR (400 MHz, acetone) δ 11.5 (s, 1H), 8.97 (dt, *J* = 2.3, 0.7 Hz, 1H), 8.29 (s, 1H), 8.26–8.18 (m, 1H), 7.84 (dd, *J* = 8.3, 0.8 Hz, 1H), 7.68–7.59 (m, 2H), 7.15–7.07 (m, 2H), 6.91 (s, 1H). ^19^F NMR (376 MHz, acetone) δ −68.06. ^13^C NMR (126 MHz, acetone) δ 169.3, 153.2, 148.5, 148.4, 145.8, 145.7 (q, *J* = 34.2 Hz), 140.3, 138.2, 135.2, 129.0, 126.9, 125.2, 123.1 (q, *J* = 272.8 Hz), 121.4 (d, *J* = 3.0 Hz), 116.1. HRMS: (ESI) [M + H]^+^ calcd for C_17_H_10_F_3_N_5_O_2_ 374.0859 observed 374.0861 *m*/*z*. Δ 0.5346

#### 6-((2′,3′-Difluoro-[1,1′-biphenyl]-4-yl)amino)-[1,2,5]oxadiazolo[3,4-*b*]pyridin-7-ol (**7as**)

Purple solid (17%, 36 mg) ^1^H NMR (400 MHz, acetone-*d*_6_) δ 10.58 (s, 1H), 8.28 (s, 1H), 7.42 (dd, *J* = 8.7, 1.8 Hz, 2H), 7.33–7.25 (m, 1H), 7.25–7.19 (m, 2H), 7.11–7.02 (m, 2H), 6.82 (s, 1H). ^19^F NMR (376 MHz, acetone *d*_6_) δ −140.5, −146.3. ^13^C NMR (126 MHz, acetone-*d*_6_) δ 169.0, 159.1, 153.2, 151.9 (dd, *J* = 245.1, 13.5 Hz), 148.5 (dd, *J* = 246.5, 13.0 Hz), 147.4, 145.6, 137.4, 132.2 (d, *J* = 10.0 Hz), 130.6 (d, *J* = 3.4 Hz), 129.6 (d, *J* = 72.4 Hz), 126.0, 125.7 (d, *J* = 8.2 Hz), 125.4 (dd, *J* = 7.5, 4.9 Hz), 118.7, 115.9 (d, *J* = 17.5 Hz), 115.7. HRMS: (ESI) [M + H]^+^ calcd for C_17_H_10_F_2_N_4_O_2_, 341.0844 observed 341.0850 *m*/*z*. Δ 1.7591 ppm.

#### 6-((2′,3′-Dichloro-[1,1′-biphenyl]-4-yl)amino)-[1,2,5]oxadiazolo[3,4-*b*]pyridin-7-ol (**7at**)

Purple solid (28%, 67 mg) ^1^H NMR (500 MHz, acetone-*d*_6_) δ 11.35 (s, 1H), 8.28 (s, 1H), 7.53 (dd, *J* = 7.9, 1.7 Hz, 1H), 7.37 (t, *J* = 7.5 Hz, 1H), 7.33 (dd, *J* = 7.7, 1.7 Hz, 1H), 7.27 (d, *J* = 8.6 Hz, 2H), 7.06 (d, *J* = 8.6 Hz, 2H), 6.78 (s, 1H). ^13^C NMR (126 MHz, acetone-*d*_6_) δ 168.8, 153.2, 147.1, 145.6, 144.0, 137.4, 133.8, 131.3, 131.0, 130.8, 130.4, 129.7, 128.7, 125.8, 115.2. HRMS: (ESI) [M – H]^−^ calcd for C_17_H_10_Cl_2_N_4_O_2_, 371.0108, observed 371.0104 *m*/*z*. Δ −1.0781 ppm.

#### 6-((3′,4′-Difluoro-[1,1′-biphenyl]-4-yl)amino)-[1,2,5]oxadiazolo[3,4-*b*]pyridin-7-ol (**7au**)

Purple solid (37%, 81 mg) ^1^H NMR (500 MHz, acetone-*d*_6_) δ 11.36 (s, 1H), 8.25 (s, 1H), 7.56–7.46 (m, 3H), 7.41 (dddd, *J* = 8.6, 4.4, 2.3, 1.3 Hz, 1H), 7.33 (dt, *J* = 10.6, 8.5 Hz, 1H), 7.07 (d, *J* = 8.7 Hz, 2H), 6.77 (s, 1H). ^19^F NMR (376 MHz, acetone-*d*_6_) δ −140.1, −143.1 to −144.9 (m). ^13^C NMR (126 MHz, acetone-*d*_6_) δ 169.1, 153.0, 151.5 (dd, *J* = 172.3, 12.8 Hz), 149.5 (dd, *J* = 172.3, 12.9 Hz), 147.0, 145.5, 139.5 (dd, *J* = 6.1, 3.7 Hz), 136.6, 130.2, 128.4, 126.0, 123.2 (dd, *J* = 6.1, 3.3 Hz), 118.3 (d, *J* = 17.2 Hz), 116.3, 115.5 (d, *J* = 17.7 Hz). HRMS: (ESI) [M – H]^+^ calcd for C_17_H_10_F_2_N_4_O_2_, 341.0847, observed 341.0853 *m*/*z*. Δ 1.7590 ppm.

#### 6-((2′,5′-Difluoro-[1,1′-biphenyl]-4-yl)amino)-[1,2,5]oxadiazolo[3,4-*b*]pyridin-7-ol (**7av**)

Purple solid (10%, 22 mg) ^1^H NMR (500 MHz, acetone-*d*_6_) δ 11.33 (s, 1H), 8.28 (s, 1H), 7.44 (dd, *J* = 8.7, 1.8 Hz, 2H), 7.32–7.17 (m, 2H), 7.08 (d, *J* = 8.8 Hz, 3H), 6.81 (s, 1H). ^19^F NMR (376 MHz, acetone-*d*_6_) δ −120.3 to −121.0 (m), −125.4 (dd, *J* = 10.2, 8.2 Hz). ^13^C NMR (126 MHz, acetone-*d*_6_) δ 159.8 (d, *J* = 239.9 Hz), 156.7 (d, *J* = 240.4 Hz), 153.1, 147.4, 145.6, 137.0, 131.5, 130.5 (d, *J* = 3.8 Hz), 125.8, 118.1 (dd, *J* = 26.5, 9.1 Hz), 116.9 (dd, *J* = 24.4, 4.2 Hz), 115.7, 115.0 (dd, *J* = 24.3, 8.7 Hz). HRMS: (ESI) [M + H]^+^ calcd for C_17_H_10_F_2_N_4_O_2_ 341.0844 observed 341.0857 *m*/*z*. Δ 3.8114 ppm.

#### 6-((3′,5′-Bis(trifluoromethyl)-[1,1′-biphenyl]-4-yl)amino)-[1,2,5]oxadiazolo[3,4-*b*]pyridin-7-ol (**7aw**)

Purple solid (44%, 127 mg) ^1^H NMR (500 MHz, acetone-*d*_6_) δ 11.42 (s, 1H), 8.29 (s, 2H), 8.19 (d, *J* = 1.8 Hz, 2H), 7.92–7.87 (m, 1H), 7.69 (d, *J* = 8.7 Hz, 2H), 7.11 (d, *J* = 8.6 Hz, 2H), 6.91 (s, 1H). ^19^F NMR (376 MHz, acetone-*d*_6_) δ −63.4. ^13^C NMR (126 MHz, acetone-*d*_6_) δ 153.2, 148.4, 145.7, 144.4, 138.0, 132.5 (d, *J* = 33.0 Hz), 129.0, 128.6, 127.0, 125.4, 124.6 (d, *J* = 272.0 Hz), 120.2, 116.1. HRMS: (ESI) [M + H]^+^ calcd for C_19_H_10_F_6_N_4_O_2_ 441.0780 observed 441.0778 *m*/*z*. Δ −0.4534 ppm.

#### 6-(Naphthalen-2-ylamino)-[1,2,5]oxadiazolo[3,4-*b*]pyridin-7-ol (**7ax**)

Purple solid (23%, 56 mg) ^1^H NMR (500 MHz, acetone-*d*_6_) δ 11.32 (s, 1H), 8.34 (s, 1H), 7.83–7.68 (m, 2H), 7.59 (dd, *J* = 8.4, 1.1 Hz, 1H), 7.38–7.32 (m, 2H), 7.30 (d, *J* = 2.3 Hz, 1H), 7.22 (ddd, *J* = 8.1, 6.8, 1.2 Hz, 1H), 6.80 (s, 1H). ^13^C NMR (126 MHz, acetone-*d*_6_) δ 168.9, 153.0, 145.4, 144.5, 135.8, 129.8, 129.5, 128.4, 127.0, 126.4, 123.5, 119.9, 109.0. HRMS: (ESI) [M + H]^+^ calcd for C_15_H_10_N_4_O_2_ 279.0877 observed 279.0878 *m*/*z*. Δ 0.3583 ppm.

#### 6-((9,9-Dimethyl-9*H*-fluoren-2-yl)amino)-[1,2,5]oxadiazolo[3,4-*b*]pyridin-7-ol (**7ay**)

Red solid (50%, 150 mg) ^1^H NMR (400 MHz, acetone-*d*_6_) δ 11.21 (s, 1H), 8.25 (s, 1H), 7.67–7.58 (m, 2H), 7.43 (d, *J* = 7.3 Hz, 1H), 7.30–7.17 (m, 3H), 7.01 (dd, *J* = 8.2, 2.1 Hz, 1H), 6.69 (s, 1H), 1.43 (s, 6H). ^13^C NMR (126 MHz, acetone-*d*_6_) δ 156.1, 153.8, 152.8, 146.1, 145.2, 140.4, 134.2, 131.9, 127.7, 127.0, 126.6, 123.2, 121.5, 119.6, 115.5, 111.2, 47.3, 27.5. HRMS: (ESI) [M + H]^+^ calcd for C_20_H_16_N_4_O_2_ 345.1346 observed 345.1345 *m*/*z*. Δ −0.2897 ppm.

#### 6-Bromo-[1,2,5]oxadiazolo[3,4-*b*]pyridin-7-ol (**8**)

6-Bromo-7-methoxy-[1,2,5]oxadiazolo[3,4-*b*]pyridine (500 mg, 2.2 mmol.) and NaOH (260 mg, 6.5 mmol.) were dissolved in dioxane (0.5 mL) and water (0.5 mL). The reaction was stirred for 15 min at room temperature. The reaction mixture was diluted with water, acidified with 10% aq. HCl and extracted with ethyl acetate (3 times). The organic extracts were combined, dried with anhydrous sodium sulfate, filtered, and concentrated *in vacuo* to afford **8** as a crude yellow amorphous solid. HRMS: (ESI) [M – H]^+^ calcd for C_6_H_4_BrN_3_O_2_, 215.9403, observed 215.9405 *m*/*z*. Δ 0.9262 ppm.

##### General Suzuki Procedure for h

To a sealed vial containing Pd(dppf)Cl_2_·CH_2_Cl_2_ (0.1 equiv), 6-bromo-[1,2,5]oxadiazolo[3,4-*b*]pyridin-7-ol (**8**) (1.00 equiv), boronic acid (1.1 equiv), and NaCO_3_ (2.5 equiv) was added 1,4-dioxane (0.1 M) and water (0.1 M) under a nitrogen atmosphere. The reaction was heated to 90 °C for 16 h. The reaction mixture was allowed to cool to room temperature, acidified with 10% aq. HCl and extracted with ethyl acetate (3 times). The organic extracts were combined, dried with anhydrous sodium sulfate, filtered, concentrated *in vacuo*, and purified via chromatography on SiO_2_ (solvent system: ethyl acetate/hexanes) to afford the desired products **9**. Yields are listed as combined over two steps.

#### 6-(2-Fluorophenyl)-[1,2,5]oxadiazolo[3,4-*b*]pyridin-7-ol (**9a**)

Yellow solid (7%, 10 mg) ^1^H NMR (400 MHz, acetone-*d*_6_) δ 11.61 (s, 1H), 8.20 (d, *J* = 0.9 Hz, 1H), 7.49 (td, *J* = 7.5, 1.8 Hz, 1H), 7.42 (dddd, *J* = 8.2, 7.2, 5.2, 1.8 Hz, 1H), 7.26 (dd, *J* = 7.5, 1.2 Hz, 1H), 7.23–7.17 (m, 1H). ^19^F NMR (376 MHz, acetone-*d*_6_) δ −111.7 to −117.6 (m). ^13^C NMR (126 MHz, acetone-*d*_6_) δ 170.4, 161.4 (d, *J* = 246.6 Hz), 153.7, 145.8, 143.7, 133.1, 130.6, 124.9, 118.5, 116.3 (d, *J* = 22.7 Hz). HRMS: (ESI) [M – H]^−^ calcd for C_11_H_6_FN_3_O_2_, 323.0516, observed 323.0526 *m*/*z*. Δ 3.0954 ppm.

#### 6-(2-Chlorophenyl)-[1,2,5]oxadiazolo[3,4-*b*]pyridin-7-ol (**9b**)

Yellow solid (20%, 45 mg) ^1^H NMR (600 MHz, acetone-*d*_6_) δ 11.64 (s, 1H), 8.12 (s, 1H), 7.51–7.50 (m, 1H), 7.40–7.39 (m, 3H). ^13^C NMR (151 MHz, acetone-*d*_6_) δ 170.3, 153.7, 145.8, 143.8, 135.3, 134.1, 133.6, 130.5, 130.2, 127.7, 122.3. HRMS: (ESI) [M + H]^+^ calcd for C_11_H_6_ClN_3_O_2_ 248.0221 observed 248.0233 *m*/*z*. Δ 4.8383 ppm.

#### 6-(3-Chlorophenyl)-[1,2,5]oxadiazolo[3,4-*b*]pyridin-7-ol (**9c**)

Yellow solid, (31%, 70 mg) ^1^H NMR (600 MHz, acetone-*d*_6_) δ 11.72 (s, 1H), 8.34 (s, 1H), 7.69 (t, *J* = 1.9 Hz, 1H), 7.58 (ddd, *J* = 7.7, 1.7, 1.0 Hz, 1H), 7.44 (t, *J* = 7.9 Hz, 1H), 7.37 (ddd, *J* = 8.0, 2.1, 1.0 Hz, 2H). ^13^C NMR (151 MHz, acetone-*d*_6_) δ 170.9, 153.5, 146.1, 143.4, 137.1, 134.3, 130.7, 129.5, 128.1, 128.1, 121.9. HRMS: (ESI) [M + H]^+^ calcd for C_11_H_6_ClN_3_O_2_ 248.0221, observed 248.0227 *m*/*z*. Δ 2.4191 ppm.

#### 6-(3-(Trifluoromethyl)phenyl)-[1,2,5]oxadiazolo[3,4-*b*]pyridin-7-ol (**9d**)

Yellow solid (26%, 67 mg) ^1^H NMR (600 MHz, acetone-*d*_6_) δ 11.75 (s, 1H), 8.42 (s, 1H), 7.99 (s, 1H), 7.90 (d, *J* = 7.4 Hz, 2H), 7.65 (m, 3H). ^19^F NMR (565 MHz, acetone-*d*_6_) δ −63.0. ^13^C NMR (151 MHz, acetone-*d*_6_) δ 171.0, 153.5, 146.1, 143.8, 136.0, 133.4, 130.7 (q, *J* = 32.0 Hz), 129.9, 126.2 (q, *J* = 3.7 Hz), 124.8 (q, *J* = 3.9 Hz), 124.4, 121.8. HRMS: (ESI) [M + H]^+^ calcd for C_12_H_6_F_3_N_3_O_2_ 282.0485 observed 282.0498 *m*/*z*. Δ 4.6446 ppm.

#### 6-(4-Chlorophenyl)-[1,2,5]oxadiazolo[3,4-*b*]pyridin-7-ol (**9e**)

Yellow solid (32%, 92 mg) ^1^H NMR (500 MHz, acetone-*d*_6_) δ 11.66 (s, 1H), 8.30 (s, 1H), 7.65 (d, *J* = 8.6 Hz, 2H), 7.44 (d, *J* = 8.6 Hz, 2H). ^13^C NMR (126 MHz, acetone-*d*_6_) δ 170.9, 153.5, 146.1, 143.0, 133.8, 133.5, 131.3, 129.0, 122.1. HRMS: (ESI) [M + H]^+^ calcd for C_11_H_6_ClN_3_O_2_ 248.0221, observed 248.0226 *m*/*z*. Δ 2.0159 ppm.

#### 6-(4-(Trifluoromethoxy)phenyl)-[1,2,5]oxadiazolo[3,4-*b*]pyridin-7-ol (**9f**)

Yellow solid (34%, 94 mg) ^1^H NMR (600 MHz, acetone-*d*_6_) δ 11.75 (s, 1H), 8.39 (s, 1H), 7.70–7.66 (m, 1H), 7.60–7.56 (m, 1H), 7.31–7.30 (m, 1H). ^19^F NMR (565 MHz, acetone-d_6_) δ −58.4. ^13^C NMR (126 MHz, acetone-d_6_) δ 170.9, 153.5, 149.8, 146.2, 143.6, 137.3, 130.7, 128.4, 122.3, 121.7, 121.5 (q, *J* = 255.5 Hz), 120.6. HRMS: (ESI) [M + H]^+^ calcd for C_12_H_6_F_3_N_3_O_3_ 298.0434 observed 298.0448 *m*/*z*. Δ 4.6973 ppm.

#### 6-(Thiophen-2-yl)-[1,2,5]oxadiazolo[3,4-*b*]pyridin-7-ol (**9g**)

Yellow solid (13%, 24 mg). ^1^H NMR (600 MHz, acetone-*d*_6_) δ 8.70 (s, 1H), 7.58 (dd, *J* = 3.7, 1.1 Hz, 1H), 7.43 (dd, *J* = 5.1, 1.2 Hz, 1H), 7.01 (dd, *J* = 5.1, 3.7 Hz, 1H). ^13^C NMR (151 MHz, acetone-*d*_6_) δ 169.3, 153.6, 145.7, 142.4, 136.0, 127.0, 125.9, 123.8, 117.4. HRMS: (ESI) [M + H]^+^ calcd for C_9_H_5_N_3_O_2_S 220.0175 observed 220.0178 *m*/*z*. Δ 1.3635 ppm.

#### 6-(4′-Fluoro-[1,1′-biphenyl]-4-yl)-[1,2,5]oxadiazolo[3,4-*b*]pyridin-7-ol (**9h**)

Yellow solid (11%, 23 mg) ^1^H NMR (400 MHz, acetone-*d*_6_) δ 11.61 (s, 1H), 8.32 (s, 1H), 7.89–7.57 (m, 6H), 7.32–7.11 (m, 2H). ^19^F NMR (376 MHz, acetone-*d*_6_) δ −117.3. ^13^C NMR (126 MHz, acetone-*d*_6_) δ 171.1, 163.4 (d, *J* = 244.7 Hz), 153.6, 146.2, 142.8, 139.7, 137.8 (d, *J* = 3.2 Hz), 134.2, 129.6 (d, *J* = 8.1 Hz), 127.4, 122.9, 116.5 (d, *J* = 21.5 Hz). HRMS: (ESI) [M + H]^+^ calcd for C_17_H_10_FN_3_O_2_ 308.0829 observed 308.0841 *m*/*z*. Δ 3.8950 ppm.

### OCR Seahorse Assay

OCR was measured using an Agilent Seahorse XF24 or XFe96 Analyzer (Agilent Technologies, Santa Clara, CA). L6 myoblasts were seeded in a Seahorse 24 or 96-well tissue culture plate at a density of 3.5 × 104 cells/well. The cells were then allowed to adhere overnight. Prior to the assay, the media was changed to unbuffered Dulbecco’s modified Eagle’s medium (DMEM) containing pyruvate and glutamine (Gibco no. 12800-017, pH = 7.4 at 37 °C), and the cells were equilibrated for 1 h at 37 °C without CO_2_. Compounds were injected during the assay, and OCR was measured using 2 min measurement periods. Cells were treated with a single drug concentration per well and measured over a 90 min period. Two wells were used per condition and, where applicable, results from multiple plates were averaged together. The first three measurements after injection for each concentration were averaged to produce a dose curve. EC_50_ values were calculated using the GraphPad Prism’s nonlinear regression built-in equation, *Y* = Bottom + (X̂Hillslope) × (Top-Bottom)/(X̂HillSlope + EC50̂HillSlope), with the Bottom constrained to the 100% baseline. AUC values were also calculated using the same software.

### Pharmacokinetics Protocol

Pharmacokinetics assessment was performed in 8-weeks-old- male C57BL/6 mice by oral gavage at a dose of 10 mg/kg of body weight. The compound was delivered in a mixture containing 99.8% (v/v) methylcellulose (0.5% solution made in water, Sigma, M0512) and 0.2% (v/v) Tween 80 (Sigma, P6474), obtained via solubilizing the drug into a Potter Elvehjem tissue homogenizer. Blood collection was performed at the indicated time points from a tail nick with a razor blade into lithium heparin-coated Microvette CB 300 tubes (Sarstedt, 16.443). Samples were processed at UNSW for liquid chromatography tandem mass spectrometry (LC–MS/MS) by protein precipitation into a mixture of acetonitrile/methanol solution (AcN-MeOH, 9:1). Seven μL of plasma was precipitated in 100 μL of AcN-MeOH, followed by centrifugation at 18,000g for 10 min and collection of the supernatant for analysis. Standards were prepared by spiking known concentrations (1–250 ng/μL) of **SHO1122147** into plasma samples from untreated mice. LC–MS/MS was performed on a Shimadzu Prominence LCMS-8030 instrument (Shimadzu, Japan). Chromatographic separation was achieved using an ACUITY UPLC BEH, C18 column (Waters, WT186002350, USA). Mobile phase A consisted of 0.1% v/v formic acid in HLPC grade water, while mobile phase B consisted of 0.1% v/v formic acid in ACN. Electrospray ionization (ESI) was performed in negative mode. Elution was achieved with a gradient of 20–100% mobile phase B at a flow rate of 0.4 mL/min with 10 μL injection volume electro-sprayed into the mass spectrometer. Transitions of *m*/*z* 336.9 > 320 and 336.9 > 336.9 with 4.29 min retention time were used. Pharmacokinetic properties were calculated using PKSolver add-in tool (Excel).

For the no observed adverse effect level (NOAEL) study, 9-week-old male C57BL/6 mice received either vehicle or **SHO1122147** (30, 100, 300, 1000 mg/kg of body weight) by oral gavage and were carefully monitored for 24 h after dosing. Core body temperature was measured with a rectal probe thermometer (Braintree, TW2–107) at the time points shown. Food intake and body weight were also recorded over time, and no signs of discomfort or ill-thrift were observed.

### Gubra-Amylin NASH (GAN) Animal Study

Animal experiments performed at UNSW were approved by the Animal Care and Ethics Committee (animal ethics approval #20/67A). C57BL/6 male mice were purchased from Australian BioResources (Moss Vale, NSW, Australia) and group-housed at 22 °C in a light-dark cycle of 12 h. Mice were provided with ad libitum access to water and normal chow diet (Gordons Specialty Feeds, NSW, Australia). We first conditioned 5/6-week-old mice on GAN diet for 33 weeks. Gubra-Amylin NASH (GAN) diet was prepared in-house and was adapted from Research Diets #D09100310: caloric content was distributed as 46% fat kcal (of which 15% were palm oil by weight), 36% sugar (of which 22% fructose and 10% sucrose by weight) and 18% protein. Diet ingredients were purchased from local suppliers. For diet preparation, we used: palm oil (MOI Foods (M) Sdn Bhd), fructose (Food Ingredients Depot, F04230), cholesterol (Sigma, C8503, 2% by weight), sucrose (JL Stewart, GRAD25B), corn starch (JL Stewart, CFLR25W), wheat bran (JL Stewart, BRAN10UF), casein (Cottee Group, NA), choline bitartrate (Sigma, C1629), lard (JL Stewart, LARD15), soybean oil (Masterol Foods, 165194538), trace minerals (MP Biomedicals, 0296026401), AIN-93 M mineral mix (MP Biomedicals, 0296040102) and AIN-93-VX vitamin mix (MP Biomedicals, 0296040201). One week before treatment, mice were single housed and stratified into groups using baseline body weight and body composition measurements to ensure similar starting parameters. Once stratified, mice were fed either GAN diet alone or GAN diet mixed with **SHO1122147** for 4 weeks; **SHO1122147** -treated mice received fresh compound daily at 200 mg/kg of body weight. Body weight and food intake were measured daily throughout the treatment period. All mice were assessed for body composition on a weekly basis during treatment period by EchoMRI. On the final day of the study, mice were anesthetized with isoflurane and exsanguinated by cardiac puncture, and harvested tissues were frozen in liquid nitrogen prior to storage at −80 °C.

### Biochemical Assays

Lipids from liver tissue were extracted using a modified version of Folch et al.’s (1957) method. Briefly, liver lipids were extracted from approximately 20 mg of tissue with 2:1 chloroform–methanol (v/v), following two washing steps of the lipid-rich lower phase in saline. Liver lipid extracts were dried under a steady stream of nitrogen in a TurboVap evaporator (Biotage) and were resuspended in 0.4 mL of 95% ethanol. Following 10 min of heating at 37 °C, lipid extracts were used to test triglycerides and cholesterol content using colorimetric assays (Pointe Scientific #T7532 and Thermo Scientific # TR13421), following manufacturer’s instructions. The results obtained were normalized to initial grams/sample extracted. Plasma alanine aminotransferase (ALT) activity was measured in ethylenediamine tetraacetic acid (EDTA) plasma samples collected at the end of the study using ALT Activity Assay (Sigma, #MAK052) following manufacturer’s instructions; samples were diluted 1:8 in ALT Assay Buffer.

### Liver Histology and NAFLD Score

Formalin-fixed liver samples were paraffin-embedded, sectioned, and stained with hematoxylin-eosin and picrosirius red. The NAFLD activity score (NAS) system was applied to all samples for scoring of steatosis, lobular inflammation and hepatocyte ballooning, and fibrosis stage was assigned as outlined by Kleiner et al., Hepatology 2005. Histological analyses were analyzed by PathCelerate Ltd. (Goostrey, Cheshire, UK); all assessments were performed by a pathologist blind to treatment.

### Statistical Analysis

All data are presented as the mean ± standard error of the mean (SEM). Statistical testing was carried out using Prism (v.10.2.0; GraphPad Software), where the threshold for significance was designated as *p* < 0.05, compared to controls. For normally distributed data, differences between groups were examined using analysis of variance (ANOVA) with Dunnett’s *post hoc* test for multiple comparisons. For nonparametric data, the Kruskal–Wallis test was conducted with Dunn’s *post hoc* test for multiple comparisons.

## Supporting Information Available

Molecular formula strings (CSV)
UPLC of **7ao**, **7ap**, **7al**, and **7m** as well as ^1^H, ^13^C NMR, and HRMS of **7m** (**SHO1122147**) (PDF)

## Abbreviations Used

ATP: adenosine triphosphate
BMI: body mass index
DNP: 2,4-dinitrophenol
GAN: Gubra-Amylin MASH
GIP: glucose dependent insulinotropic polypeptide
GLP-1: glucagon-like peptide 1
MAFLD: metabolic-associated fatty liver disease
MASH: metabolic dysfunction-associated steatohepatitis
OCR: oxygen consumption rate
PO: *per oral*
PMF: proton motive force
ROS: reactive oxygen species
SAR: structure activity relationship
UCPS: uncoupling proteins
