# Supplementary material for: Design, Synthesis, and Biological Evaluation of [1,2,5]Oxadiazolo[3,4-b]pyridin-7-ol as Mitochondrial Uncouplers for the Treatment of Obesity and Metabolic Dysfunction-Associated Steatohepatitis
Source: J Med Chem. 2024 Nov 30;67(23):21486–504. doi: 10.1021/acs.jmedchem.4c02366 (PMC11647902; doi:10.1021/acs.jmedchem.4c02366)

Supporting Information

Design, Synthesis and Biological Evaluation of [1,2,5]oxadiazolo[3,4-*b*]pyridin-7-ol as Mitochondrial Uncouplers for the Treatment of Obesity and Metabolic Dysfunction-Associated Steatohepatitis

Mary A. Foutz,[1] Emily L. Krinos,[1] Martina Beretta,[2] Stefan R. Hargett,[3] Riya Shrestha,[2] Jacob H. Murray,[1] Ethan Duerre,[1] Joseph M. Salamoun,[1] Katrina McCarter,[1] Divya P. Shah,[2] Kyle L. Hoehn,[2,3,*] Webster L. Santos[1,*]

[1]Department of Chemistry and Virginia Tech Center for Drug Discovery, Virginia Tech, Blacksburg, Virginia 24061, United States.

[2]School of Biotechnology and Biomolecular Sciences, University of New South Wales, Kensington, NSW 2033, Australia.

[3]Departments of Pharmacology and Medicine, University of Virginia, Charlottesville, Virginia 22908, United States.

*Corresponding Author Email: santosw@vt.edu, k.hoehn@unsw.edu.au

UPLC of compound 7ao, 7ap, 7al, and 7m (**SHO1122147**):..S2

^{1}H, ^{13}C NMR of **SHO1122147** S4

HRMS of **SHO1122147** S5

UPLC:

7ao

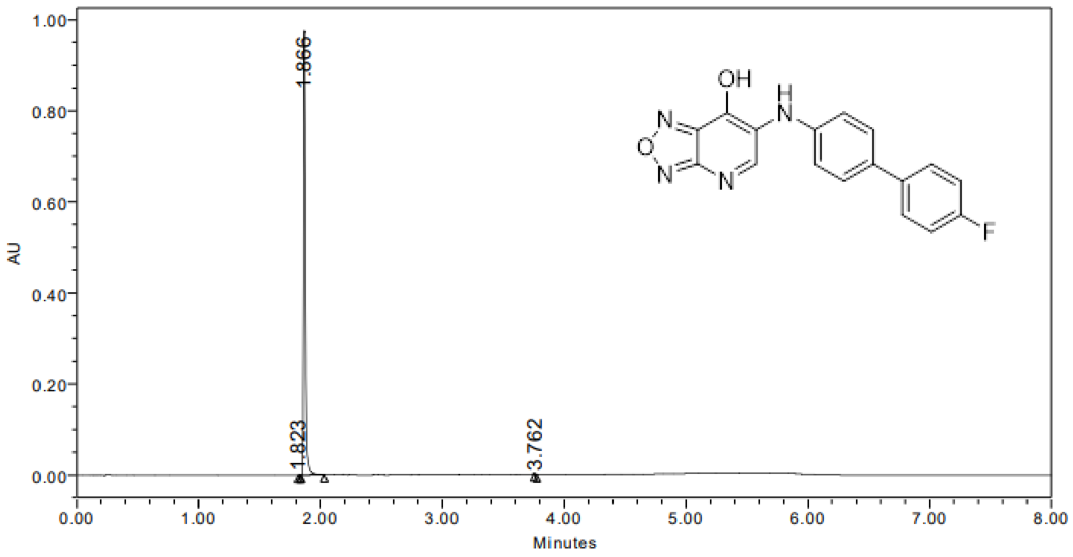

Method one used. Retention time 1.87 minutes, percent purity 99.90%

7ap

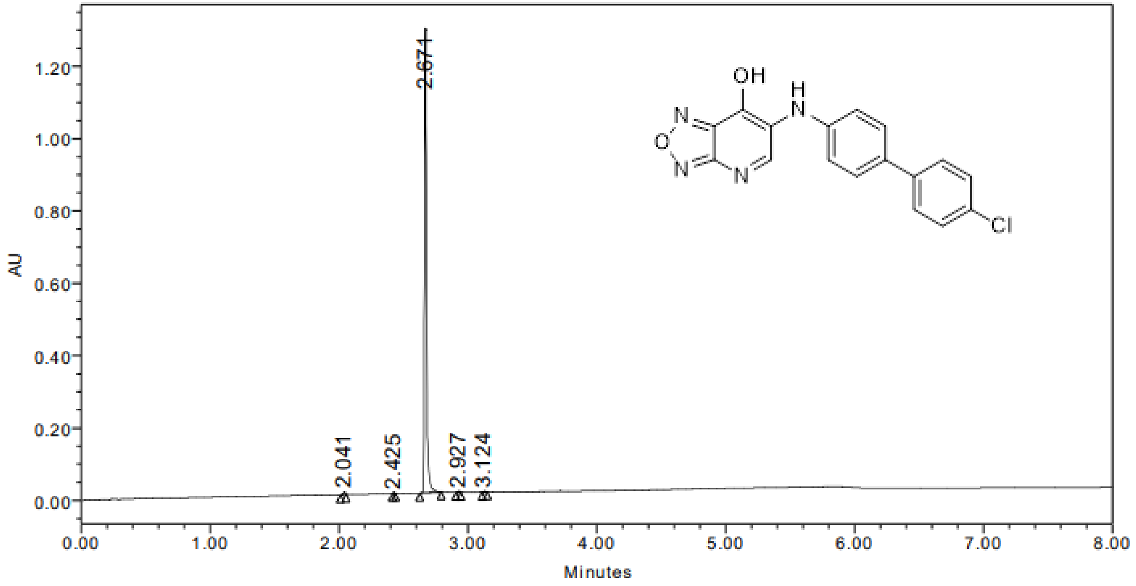

Method one used. Retention time 2.61 minutes, percent purity 99.14%

7al

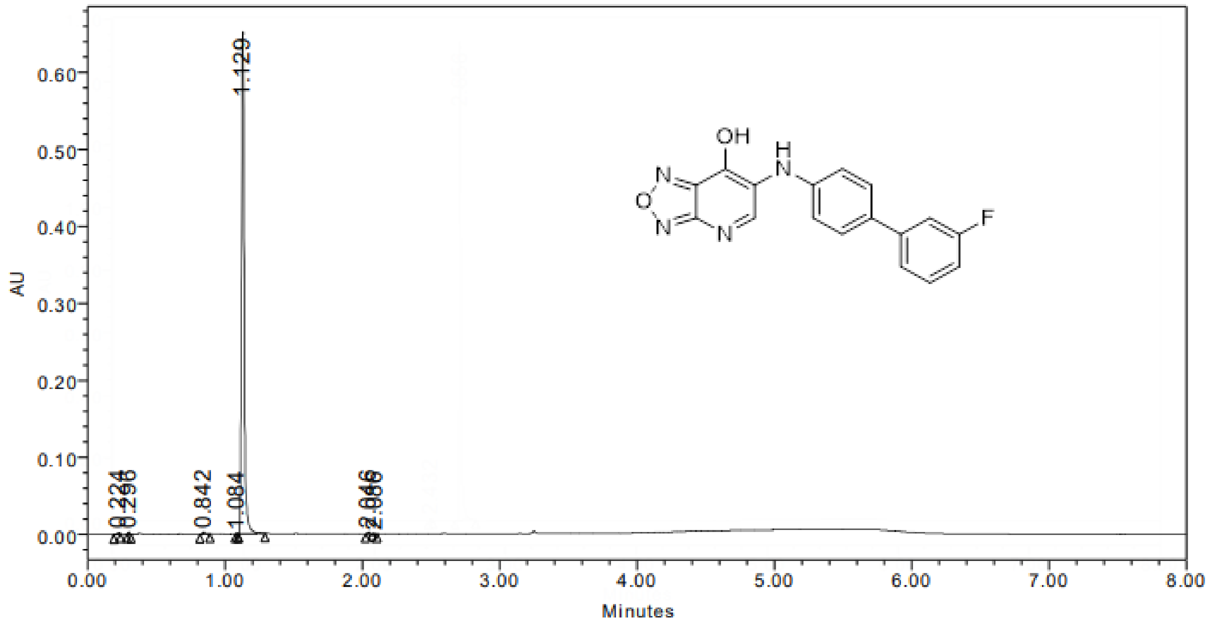

Method two used. Retention time 1.13 minutes, percent purity 98.93 %

SHO1122147

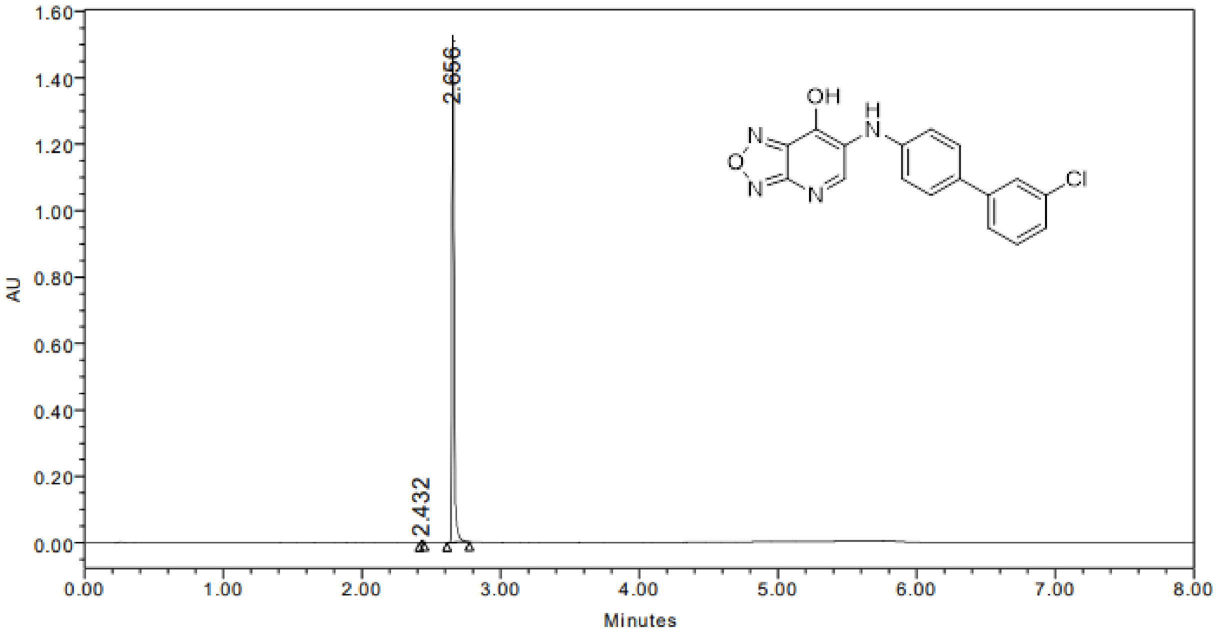

Method one used. Retention time 2.66 minutes, percent purity 99.65%

^{1}H NMR (400 MHz, Acetone-d_6) of **SHO1122147**

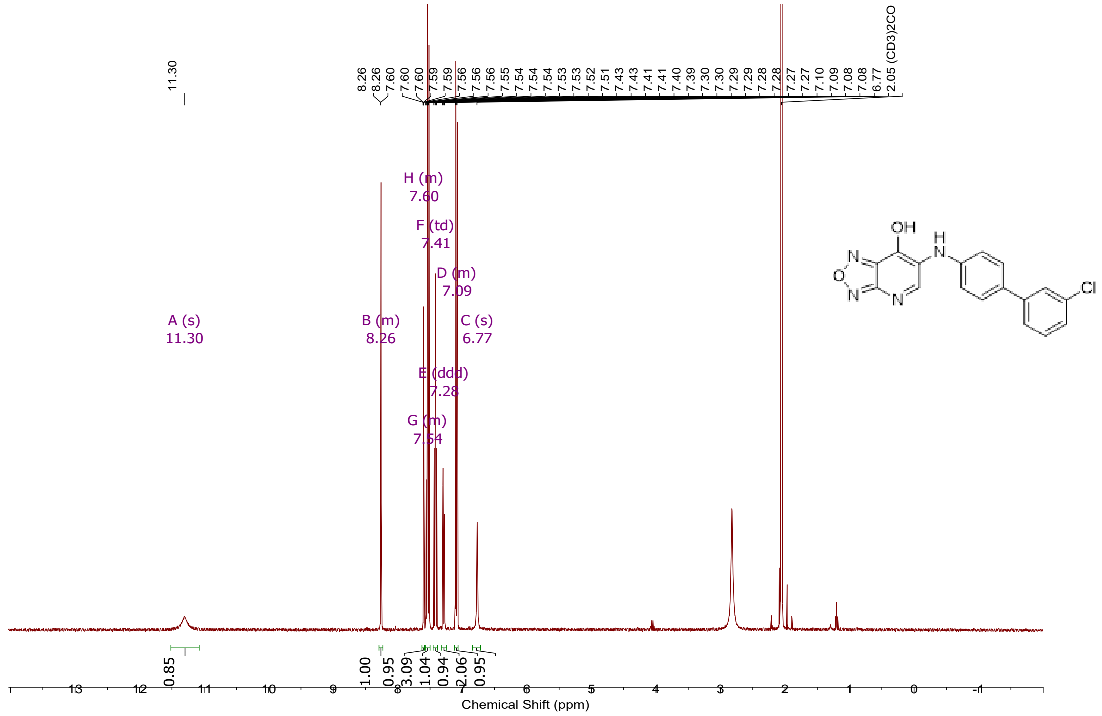

^{13}C NMR (126 MHz, Acetone-d_6) of **SHO1122147**

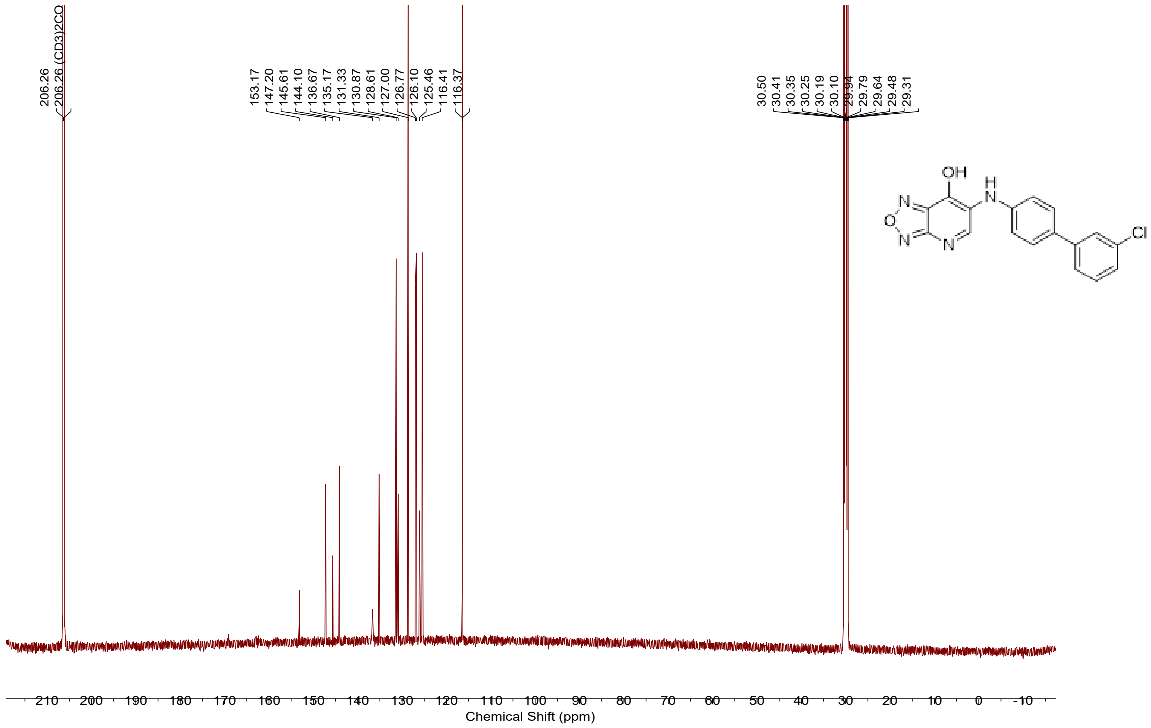

HRMS of **SHO1122147**

[illegible]

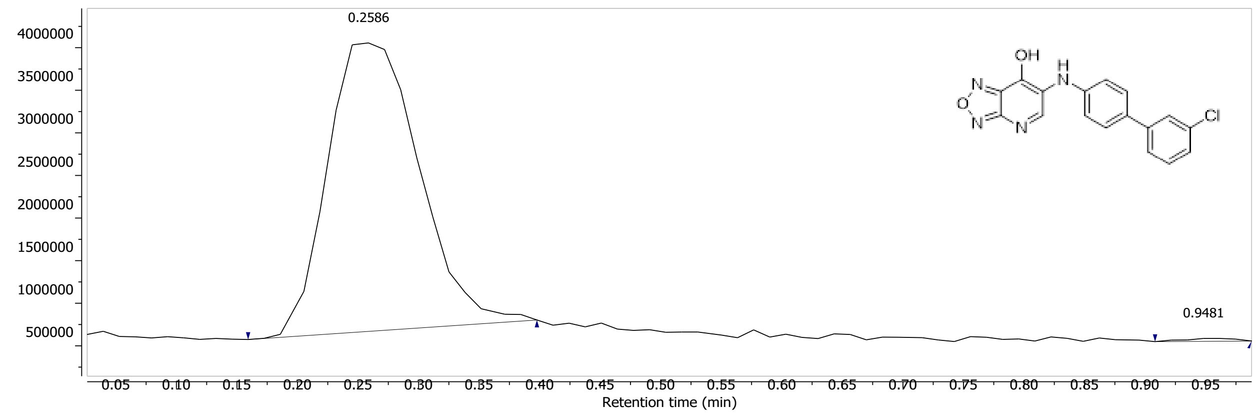

C:\Users\Joe\A...JMS_MU9-052.raw Injection 1 TOF MS ES- TOF PARENT FUNCTION MS - spectrum 0.26

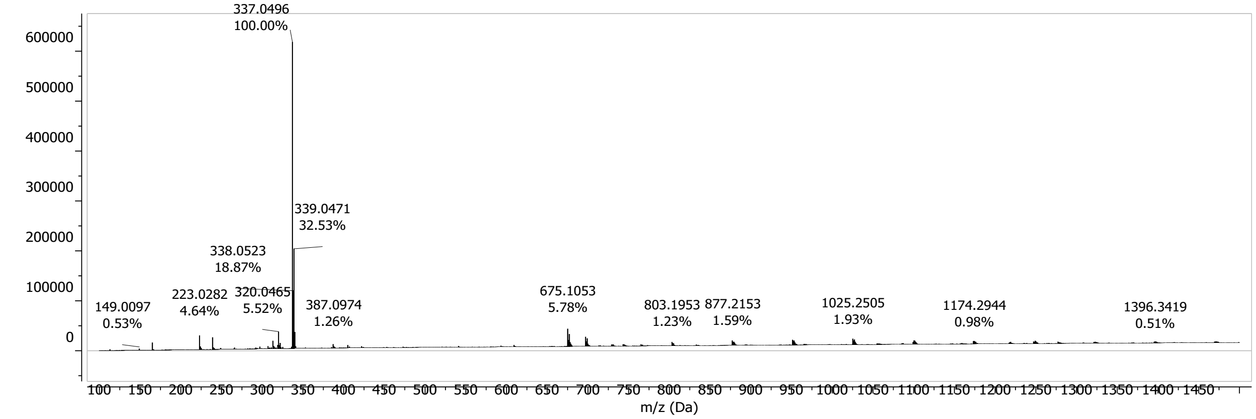

Supplement: Supplementary file 2 — jm4c02366_si_002.pdf [file jm4c02366_si_002.pdf]
